# Carbon‐Confined Fe_3_C/Fe_3_N Janus Interfaces for Selective Nitric Oxide‐to‐Ammonia Electroreduction

**DOI:** 10.1002/anie.1030679

**Published:** 2026-07-24

**Authors:** Jialing Song, Ziqi Wei, Haotian Huang, Lupeng Han, Biyi Huang, Evangelina Pensa, Sam Fong Yau Li, Eslam Hamed, Fun Man Fung, Emiliano Cortés, Dengsong Zhang

**Affiliations:** ^1^ Department of Chemistry International Joint Laboratory of Catalytic Chemistry State Key Laboratory of Materials for Advanced Nuclear Energy Innovation Institute of Carbon Neutrality College of Sciences Shanghai University Shanghai China; ^2^ Faculty of Physics Nanoinstitute Munich Ludwig‐Maximilians‐Universität (LMU) Munich Germany; ^3^ Department of Chemistry National University of Singapore Singapore Singapore; ^4^ School of Chemistry University College Dublin Dublin Ireland

**Keywords:** ammonia synthesis, Janus structure, metal carbides, metal nitrides, NO electroreduction

## Abstract

Electrocatalytic nitric oxide reduction to ammonia couples pollutant valorization with sustainable nitrogen conversion, but high activity and selectivity require concurrent control of NO transport, NO activation, and hydrogenation at the gas‐liquid‐solid interface. Here, we report a self‐supported, noble‐metal‐free Fe_3_C/Fe_3_N@C catalyst, composed of earth‐abundant Fe, C, and N, featuring defect‐rich Fe_3_C/Fe_3_N Janus nanostructures confined within graphitic carbon. The catalyst achieves an NH_3_ yield rate of 468.3 µmol h^−1^ cm^−2^ with a Faradaic efficiency of 94.2% at −0.6 V versus RHE, placing it among the most efficient reported NORR electrocatalysts. Mechanistic studies reveal that the graphitic carbon shell facilitates NO diffusion by alleviating the steric and dynamic constraints imposed by the hydrogen‐bonded water network. At the Janus interface, Fe_3_C sites preferentially adsorb and activate NO, whereas nitrogen‐vacancy‐rich Fe_3_N sites promote H_2_O dissociation to supply reactive *H for subsequent hydrogenation. This spatial coupling of mass‐transfer promotion, NO activation, and interfacial *H generation enables efficient and selective NO‐to‐NH_3_ electroreduction. These findings establish carbon‐confined, earth‐abundant carbide/nitride Janus interfaces as a promising design principle for high‐performance NORR catalysts.

## Graphical abstract

A defect‐rich, graphitic‐carbon‐confined Fe_3_C/Fe_3_N Janus catalyst integrates NO transport, adsorption/activation, and interfacial H supply for efficient nitric oxide electroreduction to ammonia. Built from earth‐abundant elements, this architecture enables high‐yield and high‐efficiency NH_3_ synthesis while valorizing industrial NO emissions.

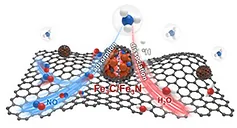

## 1 Introduction

Ammonia (NH_3_) is one of the most important commodity chemicals, used in the manufacture of fertilizers, refrigerants, and carbon‐free energy carriers [1, 2]. The primary route of industrial NH_3_ synthesis relies on the Haber‐Bosch process, which operates under harsh conditions and emits substantial amounts of CO_2_ [3, 4]. As a promising alternative, electrocatalytic reduction of nitrogen‐containing species, such as nitric oxide (NO), nitrate (NO_3_^−^), and nitrogen (N_2_), has gained increasing attention due to its green and sustainable advantages [5, 6]. The strong N≡N triple bond renders N_2_ chemically inert, posing major challenges for the N_2_ reduction reaction (NRR) [7, 8, 9]. The electroreduction of NO_3_^−^ to NH_3_ (NO_3_^−^RR) involves complex pathways and readily yields various nitrogenous by‐products [10]. In contrast, the NO reduction reaction (NORR) is more favorable than NRR due to its weaker N═O bond and higher solubility in electrolytes [11]. Moreover, NORR features a simpler reaction route and fewer side reactions than NO_3_^−^RR. Recently, NORR has garnered increasing interest, as it not only offers a promising alternative for NH_3_ synthesis but also provides an efficient means of removing NO pollutants from industrial exhaust.

However, the electrocatalytic NORR to NH_3_ is a complex reaction occurring at the gas‐liquid‐solid interface, involving NO diffusion to the catalyst surface, adsorption/activation, proton supply via H_2_O dissociation, stepwise hydrogenation, and NH_3_ product desorption [12, 13]. Due to the low solubility of NO and the steric and kinetic hindrance of the hydrogen bonding network of water, NO mass transfer limitation represents a critical and frequently neglected challenge for NORR [14, 15, 16]. Moreover, insufficient adsorption and activation of NO as well as inadequate proton supply at catalyst active sites further complicate the problem [17]. Therefore, synergistically enhancing NO diffusion, adsorption, and proton supply is key to achieving a high NH_3_ yield rate and NH_3_ Faradaic efficiency (FE) in NORR. Recently, porous carbon materials have shown potential as supports for optimizing NO transport pathways and improving the NO diffusion kinetics in water [14, 18]. In addition, most transition metals exhibit weak binding affinity toward NO and tend to form metal‐H bonds, which intensifies the competing hydrogen evolution reaction (HER) [19, 20, 21]. To overcome this limitation, materials with metal‐like electronic properties, such as metal carbides (e.g., Mo_2_C and TiC), have attracted significant attention owing to their excellent conductivity and hydrogenation activity [19, 22, 23]. Furthermore, metal nitrides with nitrogen vacancies (e.g., Cu_3_N and LaN) can effectively modulate the electronic structure of catalysts to enhance reactant adsorption and activation [24, 25, 26]. Taken together, porous carbon materials, metal carbides, and metal nitrides, provide viable platforms to improve NO diffusion, adsorption, and H_2_O dissociation. However, achieving an effective integration of their distinct catalytic functions remains a critical challenge that demands further exploration. Beyond activity and selectivity, the elemental composition of NORR catalysts is also important for practical deployment. Catalysts based on earth‐abundant elements are preferable to noble‐metal systems from the perspectives of cost, resource availability, and scalability. In this regard, iron carbides and iron nitrides are attractive platforms because they combine the abundance of Fe with metallic conductivity, tunable electronic structures, and catalytic functions relevant to NO activation and water dissociation.

In this study, a novel self‐supported defect‐rich Fe_3_C/Fe_3_N@C catalyst with a graphitic carbon‐encapsulated Fe_3_C/Fe_3_N Janus nanostructure was designed and synthesized via a top‐down electrospinning approach. Such a catalyst displayed a high NH_3_ yield rate of 468.26 µmol h^−1^ cm^−2^ with an FE of 94.18% at −0.6 V versus RHE, outperforming most reported NORR electrocatalysts in the literature. The superior NORR performance of Fe_3_C/Fe_3_N@C is attributed to the synergistic coupling of enhanced NO diffusion, adsorption and proton supply within the graphitic carbon‐encapsulated Fe_3_C/Fe_3_N Janus nanostructure. The graphitic carbon framework facilitates NO mass transfer to the catalyst surface, while the Fe_3_C/Fe_3_N Janus structure simultaneously promotes NO adsorption and proton supply via accelerated H_2_O dissociation. This study provides a viable strategy for the rational design of highly efficient NORR electrocatalysts by integrating the functions of porous carbon shells, metal carbides, and metal nitrides.

## 2 Results and Discussion

**FIGURE 1:**
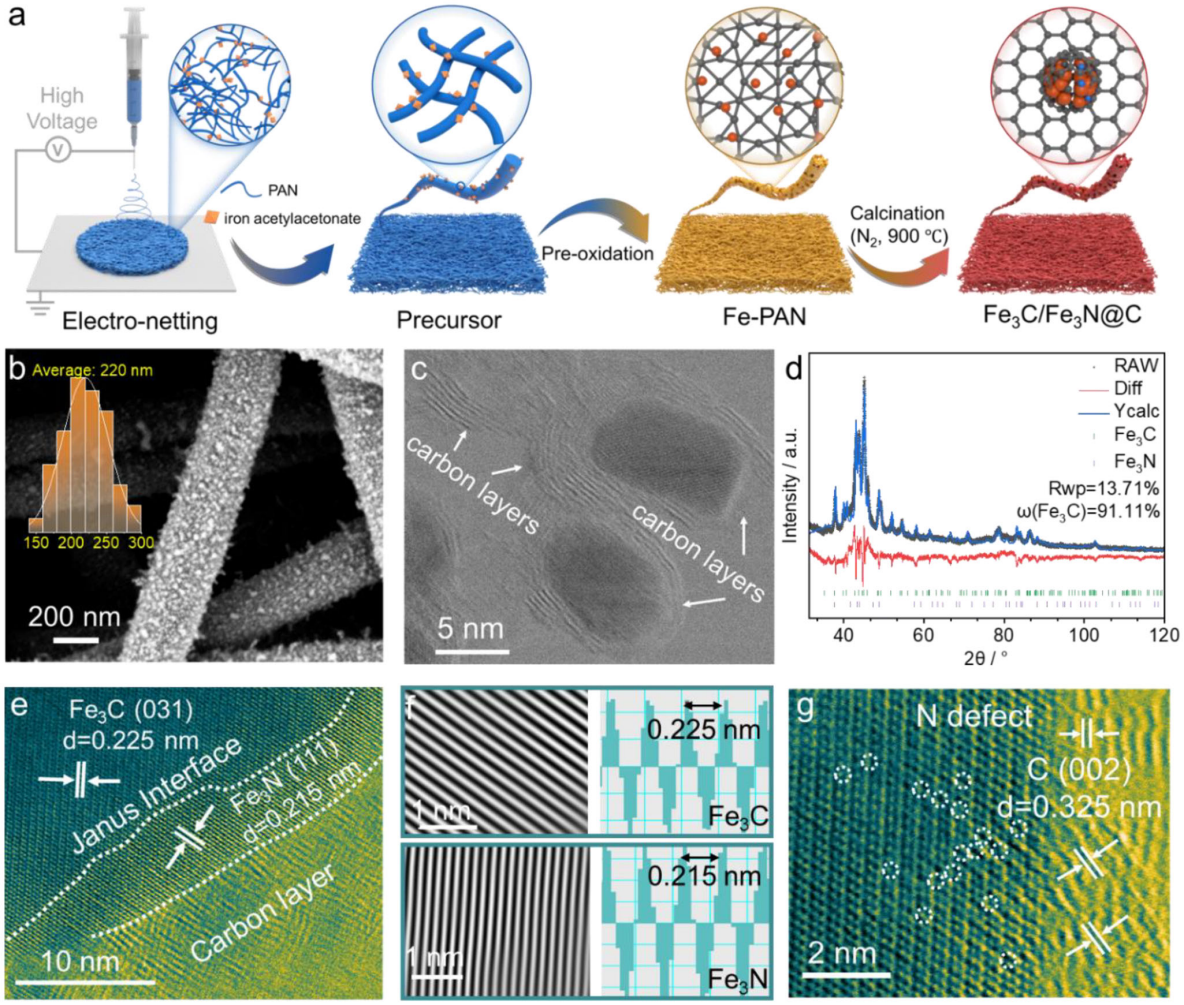
Morphology and structure of catalysts. (a) Schematic illustration of the synthesis of Fe_3_C/Fe_3_N@C. (b) SEM and (c) HR‐TEM images of Fe_3_C/Fe_3_N@C. (d) The Rietveld XRD pattern of Fe_3_C/Fe_3_N@C. (e, f) HAADF‐STEM images of Fe_3_C/Fe_3_N@C. (g) AC‐HAADF‐STEM image of Fe_3_C/Fe_3_N@C.

Figure 1a illustrates the schematic diagram of the Fe_3_C/Fe_3_N@C synthesis via a top‐down electrospinning approach. First, a self‐supported membrane was prepared by electrospinning a precursor solution containing iron acetylacetonate and polyacrylonitrile (PAN) (Figure S1). The use of PAN facilitates the introduction of nitrogen defects, as charge transfer readily occurs from PAN functional groups to metal‐ion complexes, leading to the formation of surface defects [27, 28, 29]. The self‐supported membrane was then subjected to thermal treatment at 280°C in air, undergoing cyclization and dehydrogenation to form a ladder‐like polymer structure (Fe‐PAN) [30]. Subsequently, Fe_3_C/Fe_3_N@C was obtained after the Fe‐PAN membrane was carbonized at 900°C under an N_2_ atmosphere, which promoted the formation of Fe_3_N phases through the reaction between iron and nitrogen atoms. Concurrently, the Fe_3_N phases reacted with carbon to generate Fe_3_C while simultaneously producing nitrogen vacancies. Furthermore, during high‐temperature carbonization, carbon atoms continued to reorganize around the iron‐based particles, gradually forming a graphitic carbon shell that encapsulates the surface. This shell not only stabilizes the Fe_3_C and Fe_3_N phases but also enhances the electrical conductivity and structural integrity of the composite. Scanning electron microscopy (SEM) images of Fe_3_C/Fe_3_N@C reveal that continuous and crosslinked nanofibers form a hierarchical three‐dimensional architecture with abundant nanopores (Figure 1b). The average diameter of nanofibers is approximately ∼220 nm. Interestingly, a large quantity of tiny nanotube arrays is observed on the rough carbon fiber surface with nanoparticles situated at the upper ends of the nanotubes (Figure S2). Transmission electron microscopy (TEM) revealed the morphology of graphitic carbon‐encapsulated nanoparticles (Figure 1c), and the corresponding element mappings confirmed the even distribution of Fe, C, and N within these structures (Figure S3). Rietveld‐refined x‐ray diffraction analysis (Rietveld‐refined XRD) further confirms the co‐existence of Fe_3_C and Fe_3_N, in which Fe_3_N accounts for approximately 9% of the iron sites (Figure 1d and Table S1, S2) [31]. The high‐resolution TEM image of Fe_3_C/Fe_3_N@C exhibits a distinct Janus structure, displaying lattice spacings of 0.225 nm corresponding to the (031) plane of Fe_3_C and 0.215 nm corresponding to the (111) plane of Fe_3_N (Figure 1e,f). Furthermore, the aberration‐corrected high‐angle annular dark‐field scanning transmission electron microscopy (AC‐HAADF‐STEM) of Fe_3_C/Fe_3_N@C indicates the presence of nitrogen vacancies in Fe_3_N (111) and interlayer spacing of 0.325 nm corresponding to the C (002) crystal plane (Figure 1g). As a comparison, Fe‐PAN membrane was also carbonized at 500°C (denoted as Fe_3_N@C), and the material only contained Fe_3_N phases as confirmed by XRD results (JCPDS No. 49–1662, Figure S4). In addition, due to the lower carbonization temperature, the Fe_3_N nanoparticles in Fe_3_N@C are encapsulated by amorphous carbon rather than graphitic carbon (Figure S5). Besides, Fe_3_C@C was also prepared with a similar process as Fe_3_C/Fe_3_N@C using a precursor solution containing iron acetylacetonate and polyvinylidene difluoride (without N elements). Fe_3_C@C exhibits the XRD peaks of Fe_3_C (JCPDS No. 77–0255, Figure S6) and the morphology of graphitic carbon‐encapsulated Fe_3_C nanoparticles (Figure S7). Combining the XRD and TEM results, we speculate that iron species within the nanofibers are initially converted to Fe_3_N at 500°C, which further reacts partially with carbon at 900°C to form Fe_3_C while releasing N_2_, resulting in nitrogen vacancies.

**FIGURE 2:**
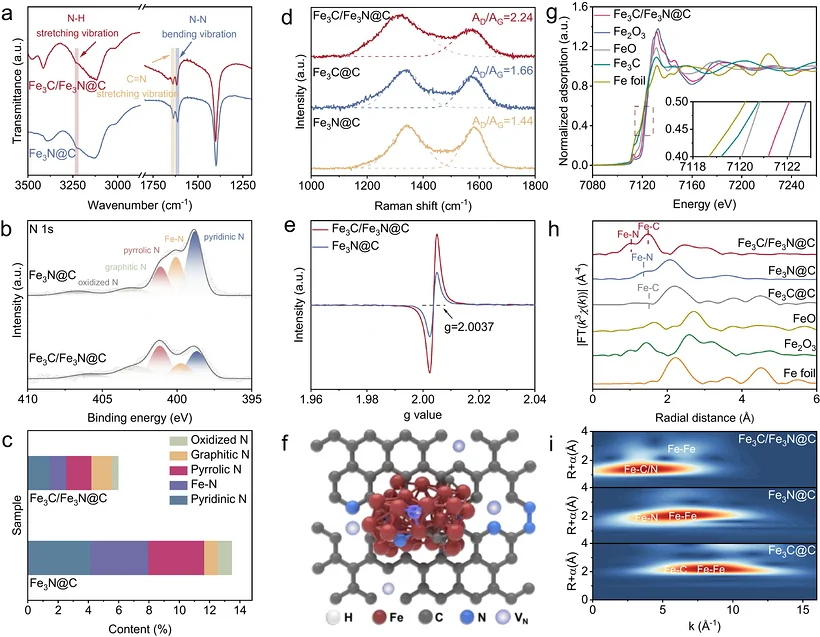
Structure analysis of catalysts. (a) FTIR spectra, (b) high‐resolution N 1s XPS spectra, (c) comparison of N species content of Fe_3_C/Fe_3_N@C and Fe_3_N@C. (d) Raman spectra of Fe_3_C/Fe_3_N@C, Fe_3_C@C, and Fe_3_N@C. (e) EPR spectra of Fe_3_C/Fe_3_N@C and Fe_3_N@C. (f) Proposed atomic structure during the pyrolysis process at 900 °C. (g) Normalized Fe K‐edge XANES spectra and (h) Fourier transform *k* ^3^‐weighted Fe K‐edge EXAFS spectra of Fe_3_C/Fe_3_N@C, Fe_3_C@C, Fe_3_N@C, Fe foil, FeO, and Fe_2_O_3_. The inset is the enlarged pre‐edge region. (i) Wavelet transforms EXAFS contour plots of Fe_3_C/Fe_3_N@C, Fe_3_C@C, and Fe_3_N@C.

Fourier transform infrared spectroscopy (FTIR) was used to analyze the structural evolution from Fe_3_N@C to Fe_3_C/Fe_3_N@C. As shown in Figure 2a, the peaks corresponding to the bending vibrations of N‐N and the stretching vibrations of N–H/C═N become less intense in Fe_3_C/Fe_3_N@C compared to Fe_3_N@C, indicating the loss of lattice N and the formation of N vacancies in Fe_3_C/Fe_3_N@C [32]. According to the x‐ray photoelectron spectroscopy (XPS) results (Figure 2b), the N content in Fe_3_C/Fe_3_N@C is significantly lower compared to that in Fe_3_N@C (Table S3 and Figure 2c), which is also consistent with the inductively coupled plasma optical emission spectrometry (ICP‐OES) results (Table S4). The N 1s spectrum can be deconvoluted into oxidized N (405.2 eV), graphitic N (402.3 eV), pyrrolic N (400.6 eV), Fe‐N (399.7 eV), and pyridinic N (398.6 eV) [33]. Generally, pyridinic N atoms located at defects or edges of the carbon matrix are more reactive due to their unsaturated coordination state [34, 35]. The pyridinic N species are preferentially eliminated in the form of N_2_ during high‐temperature carbonization, leading to the formation of nitrogen vacancies. Consequently, the pyridinic N species in Fe_3_C/Fe_3_N@C decrease notably compared to those in Fe_3_N@C. In addition, the content of Fe‐N species in Fe_3_C/Fe_3_N@C is also significantly lower than that in Fe_3_N@C. This suggests that some of the Fe‐N species in Fe_3_C/Fe_3_N@C react with carbon to form Fe_3_C and N_2_, thereby creating nitrogen vacancies. Brunauer‐Emmett‐Teller (BET) analysis shows that Fe_3_C/Fe_3_N@C has a much larger specific surface area (312.95 m^2^ g^−1^) than Fe_3_N@C (109.61 m^2^ g^−1^) (Figure S8 and Table S5), which is attributed to the existence of more defect sites. Besides, the C 1s XPS spectra confirm more graphitic carbon in Fe_3_C/Fe_3_N@C than Fe_3_N@C (Figure S9). Raman spectroscopy was also employed to probe the structural evolution of the carbon matrix [36, 37]. The characteristic D band (∼1350 cm^−1^) and G band (∼1580 cm^−1^) corresponding to lattice defects/disorder and graphitic sp^2^ carbon were observed, respectively. The ratio of the D band area to the G band area (A_D_/A_G_) serves as a key indicator of defect density within the carbon framework [38]. As shown in Figure 2d, the A_D_/A_G_ values followed a distinct order: Fe_3_C/Fe_3_N@C (2.24) > Fe_3_C@C (1.66) > Fe_3_N@C (1.44). This trend aligns with the established mechanism that intense nitrogen volatilization in nitrogen‐containing precursors at ∼900°C generates maximal lattice disorder [27]. Specifically, Fe_3_C/Fe_3_N@C undergoes the most significant nitrogen removal. This process not only etches the carbon framework to produce a high defect density but also drives the essential phase separation to form the Janus heterostructure and nitrogen vacancies. In contrast, Fe_3_N@C prepared at 500°C exhibits milder nitrogen loss and thus a lower defect density, while Fe_3_C@C derived from a nitrogen‐free polymer at 900°C lacks this disruptive volatilization pathway entirely, resulting in a more graphitized carbon support. Therefore, the elevated A_D_/A_G_ ratio in the Janus catalyst serves as a direct spectroscopic signature of the vacancy‐generating chemistry, which simultaneously constructs the active interface and optimizes the conductive carbon host for enhanced electrocatalysis. Electron paramagnetic resonance (EPR) spectroscopy further confirms that Fe_3_C/Fe_3_N@C possesses more nitrogen vacancies than Fe_3_N@C (Figure 2e), as evidenced by the stronger signal intensity at *g* = 2.003 that is widely reported for nitrogen vacancies in carbon‐based metal nitrides and N‐doped carbons [24, 39, 40]. Based on the above results, the proposed atomic structures during the thermal tuning process at 500°C and 900°C are illustrated in Figure 2f and Figure S10, respectively.

The chemical states and local environments of Fe atoms in Fe_3_C/Fe_3_N@C were evaluated using x‐ray absorption near‐edge structure (XANES) and extended x‐ray absorption fine structure (EXAFS) spectroscopy. The Fe K‐edge adsorption position of Fe_3_C/Fe_3_N@C lies between those of FeO and Fe_2_O_3_, indicating an average valence state of Fe between +2 and +3 (Figure 2g). The edge energy of Fe_3_C/Fe_3_N@C is higher than that of the Fe_3_C reference, suggesting a higher Fe valence state in Fe_3_C/Fe_3_N@C, in line with Fe 2p XPS results (Figure S11). This shift is ascribed to the incorporation of Fe_3_N, where the more electronegative N (relative to C) withdraws more electron density from Fe. To elucidate the coordination environment of Fe atoms in these samples, the EXAFS spectra were Fourier‐transformed into R‐space (Figure 2h). Fe_3_N@C displays Fe‐N (1.36 Å) and Fe‐Fe (2.05 Å) bonds, while Fe_3_C@C shows Fe‐C (1.60 Å) and Fe‐Fe (2.10 Å) bonds, respectively. In comparison, Fe_3_C/Fe_3_N@C exhibits a significantly intensified peak at ∼1.48 Å, which is attributed to the superimposed scattering from Fe‐N and Fe‐C bonds. The FT‐EXAFS spectra of Fe_3_C/Fe_3_N@C, Fe_3_N@C, and Fe_3_C@C can be well‐fitted using the theoretical structure models (Figures S12–S15, Table S6). Quantitative fitting reveals a total first‐shell Fe coordination number of ∼3.8 (from Fe‐N and Fe‐C), substantially higher than the values for the single‐phase references of Fe‐N (2.0) in Fe_3_N@C and Fe‐C (1.4) in Fe_3_C@C, thus confirming the coexistence of Fe‐N and Fe‐C bonds in Fe_3_C/Fe_3_N@C [41, 42]. In addition, Fe_3_C/Fe_3_N@C shows a lower Fe‐Fe coordination number (4.3) than Fe_3_N@C (10.2) and Fe_3_C@C (7.3), which indicates a higher dispersion of Fe atoms and, consequently, an increased number of accessible active sites. The wavelet transform (WT) contour plot of Fe_3_C/Fe_3_N@C further displays overlapping signals of Fe‐N and Fe‐C relative to those of Fe_3_N@C and Fe_3_C@C (Figure 2i, Figures S16–S18). Collectively, the above results demonstrate that Fe_3_C/Fe_3_N@C features a Fe_3_C/Fe_3_N Janus structure encapsulated by graphitic carbon.

**FIGURE 3:**
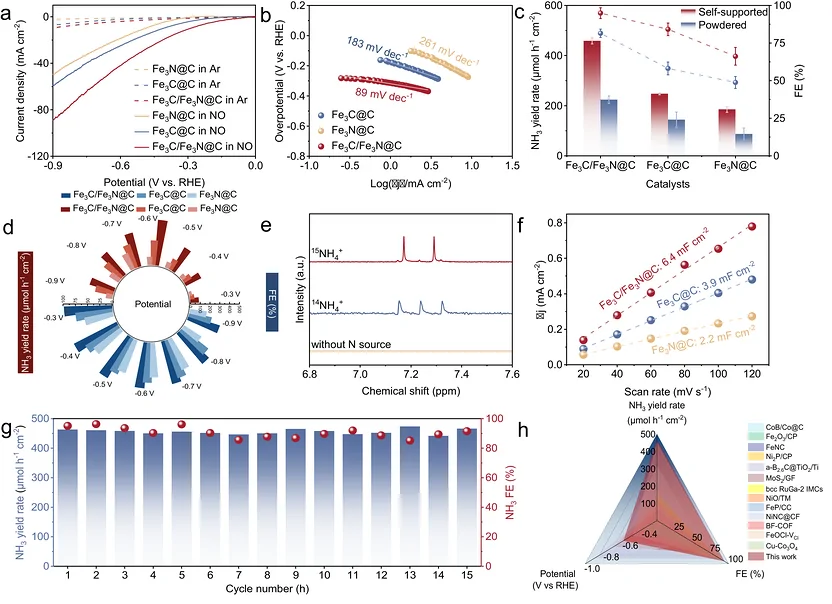
NORR performance. (a) LSV curves of Fe_3_C/Fe_3_N@C, Fe_3_C@C, and Fe_3_N@C with a scanning rate of 5 mV s^−1^ recorded in 0.5 M Ar‐saturated and NO‐saturated Na_2_SO_4_ (with 90% iR‐correction). (b) Tafel slopes of Fe_3_C/Fe_3_N@C, Fe_3_C@C, and Fe_3_N@C. (c) NH_3_ yield rate and FE on Fe_3_C/Fe_3_N@C, Fe_3_C@C, and Fe_3_N@C. (d) The comparison of NH_3_ yield rate and FE for Fe_3_C/Fe_3_N@C, Fe_3_C@C, and Fe_3_N@C at different applied potentials. (e) NMR spectra of isotopic labeling experiments on Fe_3_C/Fe_3_N@C. (f) The C_dl_ comparison of Fe_3_C/Fe_3_N@C, Fe_3_C@C, and Fe_3_N@C. (g) Cycle stability tests of Fe_3_C/Fe_3_N@C at −0.6 V versus RHE. Each cycle requires a replacement of the 0.5 M Na_2_SO_4_ electrolyte. (h) Comparison of NH_3_ electrosynthesis performance of Fe_3_C/Fe_3_N@C with previously reported electrocatalysts.

The electrocatalytic NORR measurements of all the catalysts were conducted in NO‐saturated 0.5 M Na_2_SO_4_ electrolyte using a gastight H‐type cell. Prior to introducing NO, the electrolyte was purged with high‐purity argon for 30 min to eliminate oxygen from the system. The linear sweep voltammetry (LSV) curves (Figure 3a) show that Fe_3_C/Fe_3_N@C exhibits notably higher current in the NO‐saturated electrolyte than in the Ar‐saturated electrolyte, and its current is also larger than those of Fe_3_N@C and Fe_3_C@C, indicating the superior NORR activity of Fe_3_C/Fe_3_N@C. Tafel slope analysis (Figure 3b) further revealed an earlier current response and smaller slopes for Fe_3_C/Fe_3_N@C, suggesting that the introduction of nitrogen vacancies and the Janus structure enhanced its NORR kinetics. The NH_3_ yield was determined by the indophenol blue spectrophotometric (IBS) method with the corresponding calibration curve (Figure S19). As shown in Figure 3c, the self‐supported Fe_3_C/Fe_3_N@C, Fe_3_C@C, and Fe_3_N@C catalysts outperform their powdered counterparts at −0.6 V versus RHE. This enhanced performance can be attributed to the integrated conductive network, where the in‐situ formed carbon shells promote efficient inter‐component electron transfer, while the binder‐free 3D architecture maximizes the exposure of active sites and facilitates NO diffusion. As shown in Figure 3d, the NH_3_ yield and FE of Fe_3_N@C, Fe_3_C@C, and Fe_3_C/Fe_3_N@C display a volcano‐shaped curve from −0.3 to −0.9 V. When the potential increased from −0.3 to −0.6 V, the NH_3_ yield and FE gradually increased due to the accelerated reaction kinetics of NORR and a higher *H utilization rate during the hydrogenation steps. However, when the applied potential exceeds −0.6 V versus RHE, the NH_3_ yield rate and FE decline due to competing HER. Among the three catalysts, Fe_3_C/Fe_3_N@C achieves the highest NH_3_ yield of 468.26 µmol h^−1^ cm^−2^ and FE of 94.18% at −0.6 V versus RHE. The possible liquid byproducts hydroxylamine (NH_2_OH) and hydrazine hydrate (N_2_H_4_) were not detected during the NORR process (Figures S20–S23). A trace amount of H_2_ was detected, while N_2_ and N_2_O were not observed by differential electrochemical mass spectrometry (DEMS), establishing H_2_ as the primary NORR by‐product (Figure S24). Notably, the relative intensity of the H_2_ signal follows the order Fe_3_C/Fe_3_N@C < Fe_3_C@C < Fe_3_N@C (Figure S25), consistent with the progressively decreasing NH_3_ selectivity. This trend is further supported by gas chromatography (GC) results, which show corresponding Faradaic efficiencies for H_2_ of 2.55% (Fe_3_C/Fe_3_N@C), 12.83% (Fe_3_C@C), and 28.75% (Fe_3_N@C), respectively (Figure S26). The NH_3_ yield and FE determined by ^1^H nuclear magnetic resonance (NMR) show similar product concentrations to those obtained from UV‐vis measurements, demonstrating the accuracy of the quantitative analysis in this work (Figures S27–S28). Subsequently, a series of control experiments were conducted to determine the nitrogen source of the produced NH_3_. Trace amounts of NH_3_ products were detected in the NO‐saturated electrolyte without an applied potential and in the Ar‐saturated electrolyte with an applied potential, thus eliminating possible interference from nitrogen‐containing contaminants (Figure S29). To further confirm the origin of the generated NH_3_, isotopic labeling experiment using ^15^NO and ^14^NO were performed and analyzed by ^1^H NMR. As shown in Figure 3e, the NMR spectrum exhibits a characteristic ^14^NH_4_^+^ triplet signal with a coupling constant of 52 Hz when ^14^NO is used as the N precursor, whereas a doublet signal with a coupling constant of 72 Hz associated with ^15^NH_4_^+^ appears when ^15^NO is used. These results demonstrate that the generated NH_3_ originates from the electroreduction of NO rather than from any nitrogen‐containing impurities in the electrolytic system.

To evaluate the catalytic activity of the catalysts, we calculated the double‐layer capacitances (*C*_dl_) and accessed the electrochemical active surface area (ECSA) based on cyclic voltammetry (CV) curves at scan rates ranging from 10 to 100 mV s^−1^ (Figure 3f and S30). Fe_3_C/Fe_3_N@C exhibits a higher C_dl_ (6.4 mF cm^−2^) than those of Fe_3_C@C (3.9 mF cm^−2^) and Fe_3_N@C (2.2 mF cm^−2^), indicating a larger exposed catalytic surface during NORR. Besides, the turnover frequency (TOF) value of Fe_3_C/Fe_3_N@C was calculated to be 0.59 s^−1^ at −0.6 V versus RHE, which is higher than those of Fe_3_N@C (0.46 s^−1^) and Fe_3_C@C (0.51 s^−1^) (Figure S31). These results suggest that Fe_3_C/Fe_3_N@C exhibits higher intrinsic activity than Fe_3_N@C and Fe_3_C@C. Electrochemical impedance spectroscopy measurement (EIS) (Figure S32) illustrates that Fe_3_C/Fe_3_N@C exhibits the smallest charge transfer resistance (2.62 Ω) compared to those of Fe_3_N@C (4.68 Ω) and Fe_3_C@C (5.08 Ω), indicating faster electron transfer kinetics during catalysis. Moreover, the NH_3_ yield and FE of Fe_3_C/Fe_3_N@C do not show significant fluctuation after 15 cycles (Figure 3g), and the system also retained its original current density during a 100‐h test, demonstrating the exceptional electrochemical stability of Fe_3_C/Fe_3_N@C (Figure S33). TEM, XRD, and XPS analysis after the stability tests reveal that the crystal structure and chemical composition of Fe_3_C/Fe_3_N@C remain unchanged, demonstrating its structural stability (Figures S34–S38). It is worth noting that Fe_3_C/Fe_3_N@C is superior to most reported electrochemical NORR catalysts in terms of NH_3_ yield, FE, and applied potential (Figure 3h and Table S7). In addition, we assembled a proof‐of‐concept Zn‐NO battery, which can simultaneously eliminate nitrogen oxides, generate NH_3_, and output electrical energy [43]. Surprisingly, the calculated maximum power density of the Zn‐NO battery is 16.28 mW cm^−2^, and the NH_3_ yield rate is 519.8 µg h^−1^ cm^−2^ at 8 mA cm^−2^ (Figure S39 and Table S8), showing great potential for practical applications.

**FIGURE 4:**
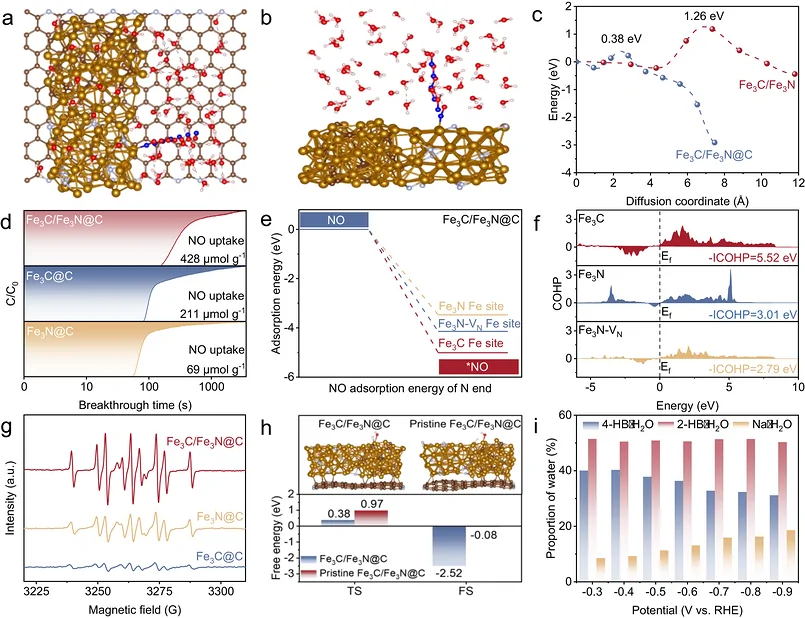
NORR mechanism. Simulated NO diffusion path in the electrolyte with (a) Fe_3_C/Fe_3_N@C and (b) Fe_3_C/Fe_3_N as the catalysts. (c) Corresponding NO migration energy barriers of Fe_3_C/Fe_3_N@C and Fe_3_C/Fe_3_N. (d) In situ NO adsorption breakthrough curves of Fe_3_C/Fe_3_N@C, Fe_3_C@C, and Fe_3_N@C. (e) The calculated NO adsorption energy of N‐end mode on Fe sites within Fe_3_C, Fe_3_N, and Fe_3_N‐V_N_. (f) The COHP analysis of Fe‐N bond during NO adsorption on Fe_3_C, Fe_3_N, and Fe_3_N‐V_N_ sites within Fe_3_C/Fe_3_N@C. (g) EPR spectra recorded on Fe_3_C/Fe_3_N@C, Fe_3_C@C, and Fe_3_N@C upon electrolysis in the absence of NO. (h) Gibbs free energy for H_2_O dissociation on Fe_3_C/Fe_3_N@C with/without N vacancies. (i) Potential‐dependent population of interfacial water on Fe_3_C/Fe_3_N@C.

To elucidate the reasons behind the excellent NORR performance of Fe_3_C/Fe_3_N@C, a series of computational studies were conducted. Efficient NORR is often limited by the diffusion of NO molecules to the catalyst surface, a process often hindered by the hydrogen‐bonded network of surrounding water molecules [23, 44]. Using density functional theory (DFT), the migration pathway of NO molecules in solution was simulated for Fe_3_C/Fe_3_N Janus nanoparticles with and without carbon encapsulation (Figure 4a,b). For Fe_3_C/Fe_3_N nanoparticles without carbon encapsulation, the hydrogen‐bonded network of water imposes steric and dynamic constraints that force NO to navigate through narrow interstitial spaces, resulting in a convoluted migration pathway and significant mass transport limitation (Figure 4b). In contrast, when the Fe_3_C/Fe_3_N nanoparticles are encapsulated by a graphitic carbon shell, the carbon surface attracts and accumulates NO molecules, forming a more direct and unobstructed diffusion route toward the active sites at the solid‐liquid interface (Figure 4a). DFT‐calculated diffusion energies further confirm this mechanism: the maximum migration energy barrier decreases from 1.26 eV without carbon capsulation to only 0.38 eV with it (Figure 4c). This “carbon‐bridge” effect not only enhances NO mass transport and increases its surface coverage, which facilitates the overall NORR process, but it also suppresses the competing HER by limiting H‐H coupling, thereby improving the FE for NH_3_ production.

Once NO molecules overcome the diffusion barrier and reach the catalyst surface, the next crucial step for efficient NORR is the adsorption and activation of NO at the active sites. The NO adsorption capacities of the three catalysts were first explored using in‐situ gas adsorption breakthrough tests (Figure 4d). The NO breakthrough curve of Fe_3_C/Fe_3_N@C displays a longer saturation time compared to those of Fe_3_N@C and Fe_3_C@C, and quantitative analysis shows that the NO adsorption amount of Fe_3_N@C, Fe_3_C@C, and Fe_3_C/Fe_3_N@C is 69, 211, and 428 µmol g^−1^, respectively. These results suggest that Fe_3_C sites inherently favor NO capture more effectively than Fe_3_N sites. To elucidate the thermodynamic origin of this behavior, DFT calculations were performed on Fe_3_C, Fe_3_N, and N‐vacancy‐containing Fe_3_N (Fe_3_N‐V_N_) sites within the heterostructure (Figure 4e and S40). The N‐end adsorption mode is more favorable than the O‐end mode due to its more negative adsorption energy on Fe_3_C (−5.01 vs. −3.91 eV, Figure S41). After structure optimization, the N‐end adsorption energies of NO on Fe_3_C, Fe_3_N, and Fe_3_N‐V_N_ are −5.01, −3.7, and −4.14 eV, respectively. The stronger adsorption on Fe_3_C arises from the higher electron‐donating ability of C atoms, which increases the electron density at Fe, strengthens π back‐donation to NO, and facilitates its activation. Notably, while Fe_3_C offers the strongest individual adsorption affinity, the NO adsorption capacity of the Fe_3_C/Fe_3_N@C heterostructure (428 µmol g^−1^) is further governed by geometric factors. As confirmed by BET analysis (Table S5), Fe_3_C/Fe_3_N@C possesses a much larger specific surface area (312.95 m^2^ g^−1^) and pore volume (0.28 cm^3^ g^−1^) compared to Fe_3_C@C (233.08 m^2^ g^−1^ and 0.18 cm^3^ g^−1^, respectively). This increased surface area and pore volume ensure a higher density of accessible active sites, thereby maximizing the total NO adsorption capacity of Fe_3_C/Fe_3_N@C. Further insights from crystal orbital Hamilton population (COHP) analysis reveal that Fe_3_C exhibits a more negative integrated COHP (ICOHP) value for the Fe‐N bond (−5.52 eV) than Fe_3_N (−3.01 eV) and Fe_3_N‐V_N_ (−2.79 eV), confirming the stronger Fe‐N bond on Fe_3_C (Figure 4f and Figure S42). Overall, these findings demonstrate that Fe_3_C sites in Fe_3_C/Fe_3_N@C serve as the primary NO adsorption centers, while the presence of Fe_3_N‐V_N_ sites further improves NO adsorption capability.

Following efficient NO adsorption and activation at the catalyst surface, the availability and reactivity of surface‐bound hydrogen (*H) play a crucial role in driving the hydrogenation and stepwise conversion of NO intermediates toward NH_3_ formation [45, 46]. As a result, the catalysts’ ability to dissociate H_2_O, which governs the *H supply, was investigated. EPR spectroscopy was first used to probe *H formation in Na_2_SO_4_ electrolyte (Figure 4g). The observed DMPO‐H signal with an intensity ratio close to 1:1:2:1:2:1:2:1:1 matches the calculated hyperfine coupling constants (*a*_N_ = 16.5 G and *a*_H_ = 21.3 G). The overall EPR signal intensities reveal that Fe_3_C@C exhibits limited *H generation, Fe_3_N@C demonstrates significantly improved *H generation, and Fe_3_C/Fe_3_N@C displays the strongest *H generation ability among the three. These results suggest that the Fe_3_N sites within Fe_3_C/Fe_3_N@C serve as the primary sites for H_2_O dissociation to produce *H. To explore the role of nitrogen vacancies in boosting *H supply, DFT calculations were employed to model Fe_3_C/Fe_3_N@C with (Fe_3_C/Fe_3_N@C) or without (Pristine Fe_3_C/Fe_3_N@C) N vacancies for comparison (Figures S43, S44). The calculated transition state (TS) activation energy barriers for H_2_O dissociation on Fe_3_C/Fe_3_N@C and Fe_3_C/Fe_3_N‐NV@C are 0.38 eV and 0.97 eV, respectively (Figure 4h and Figure S45), indicating that the presence of N vacancies makes H_2_O dissociation more kinetically favorable and thus enhances *H generation for the NORR process. To further investigate the H_2_O dissociation ability of Fe_3_C/Fe_3_N@C, in‐situ Raman spectroscopy measurements were conducted under various applied potentials. The interfacial water species consist of 4‐coordinated hydrogen‐bonded water (4‐HB·H_2_O), 2‐coordinated hydrogen‐bonded water (2‐HB·H_2_O), and Na^+^ ion‐hydrated water (Na·H_2_O). Among these, Na·H_2_O represents weakly hydrogen‐bonded and more easily dissociable water molecules that act as key *H sources during NORR [47, 48, 49]. Therefore, we performed Gaussian fitting of the broad O‐H stretching band from 2800 to 3800 cm^−1^, and three distinct peaks corresponding to 4‐HB·H_2_O, 2‐HB·H_2_O, and Na·H_2_O were resolved from low to high wavenumbers [50]. As shown in Figure 4i, increasing the negative potential results in a substantial growth of Na·H_2_O content on Fe_3_C/Fe_3_N@C (from 8.53% to 18.55%), which is much higher than that of Fe_3_C@C (from 6.44% to 10.49%) and Fe_3_N@C (from 4.94% to 12.59%) (Figures S46–S50). This trend demonstrates the superior ability of Fe_3_C/Fe_3_N@C to activate water and generate *H. Overall, the synergistic design of Fe_3_C/Fe_3_N@C enables efficient H_2_O dissociation and abundant H* production, which, combined with its enhanced NO diffusion, adsorption, and activation capabilities, ensures fast and continuous NO hydrogenation while simultaneously suppresses competing HER [49].

**FIGURE 5:**
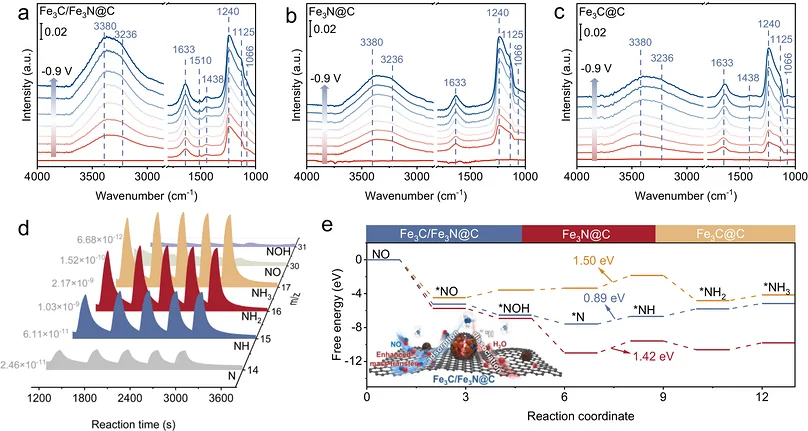
Reaction pathway and mechanism. In situ ATR‐SEIRAS spectra recorded on (a) Fe_3_C/Fe_3_N@C, (b) Fe_3_N@C, and (c) Fe_3_C@C in 0.5 M Na_2_SO_4_ at applied potentials ranging from OCP to −0.9 V versus RHE. (d) Online DEMS measurements on Fe_3_C/Fe_3_N@C at −0.6 V versus RHE. (e) The calculated NORR Gibbs free energy diagrams on Fe_3_C/Fe_3_N@C, Fe_3_N@C, and Fe_3_C@C.

To clarify the possible reaction intermediates and pathway of NORR, in‐situ attenuated total reflection surface‐enhanced infrared absorption spectroscopy (ATR‐SEIRAS) measurements were conducted in the potential range from OCP to −0.9 V (vs. RHE). With increasing negative potential, characteristic peaks corresponding to NH_4_^+^ (1438 cm^−1^), NH_3_ (1633 and 1240 cm^−1^), N–H bending modes (1510 and 1066 cm^−1^), and *HNO (1125 cm^−1^) species become more intense on Fe_3_C/Fe_3_N@C (Figure 5a) [51, 52]. Meanwhile, a broad band ranging from 3100 to 3700 cm^−1^, attributed to O–H species, increases in intensity, demonstrating the consumption of protons during NO hydrogenation [53, 54]. The time‐resolved in situ ATR‐SEIRAS spectra further confirm the generation of these reaction intermediates on Fe_3_C/Fe_3_N@C (Figure S51). For both Fe_3_N@C (Figure 5b) and Fe_3_C@C (Figure 5c), similar peaks corresponding to NH_3_, N‐H bending modes, *HNO, and O‐H species are also observed, but their intensities remain significantly weaker, especially at less negative potentials, thus implying their lower capacities for NO adsorption, activation, and subsequent hydrogenation compared to Fe_3_C/Fe_3_N@C.

To further confirm the key reaction intermediates, differential electrochemical mass spectrometry (DEMS) measurements were performed at −0.6 V versus RHE. As shown in Figure 5d, signals corresponding to *NOH, *N, *NH, *NH_2_ and *NH_3_ are detected on Fe_3_C/Fe_3_N@C. The NO signal gradually decreases as the reaction proceeds, demonstrating continuous consumption of NO during NORR (Figure S52). The intermediates observed in DEMS agree with those identified by in‐situ ATR‐SEIRAS measurements. Based on these results, the NORR pathway on Fe_3_C/Fe_3_N@C is proposed to follow a step‐by‐step hydrogenation process: NO → *NO → *NOH → *N → *NH → *NH_2_ → *NH_3_ → NH_3_. The reaction energy diagrams of NORR for Fe_3_C/Fe_3_N@C, Fe_3_N@C, and Fe_3_C@C are displayed in Figure 5e (the structural models of intermediates are shown in Figures S53–S55). The initial hydrogenation of NO preferentially forms *NOH rather than *HNO, while the second hydrogenation of NOH* to N* is more favorable than its hydrogenation to HNOH* (Figure S56). The hydrogenation of *N to *NH is the rate‐determining step (RDS) for all catalysts, with an uphill free energy of 0.89, 1.42, 1.50 eV on Fe_3_C/Fe_3_N@C, Fe_3_N@C, and Fe_3_C@C, respectively. Besides, for Pristine Fe_3_C/Fe_3_N@C (Figures S57–S58), the *NO → *NOH and *NH → *NH_2_ steps exhibit much higher energy barriers of 2.67 and 2.70 eV, respectively, indicating that nitrogen vacancies play a vital role in facilitating these transformations. Collectively, these findings demonstrate that the conversion of NO to NH_3_ is thermodynamically and kinetically more favorable on Fe_3_C/Fe_3_N@C with nitrogen vacancies than on Fe_3_C@C, Fe_3_N@C, and Fe_3_C/Fe_3_N@C without nitrogen vacancies. Based on the above analysis, the NORR reaction mechanism of Fe_3_C/Fe_3_N@C is illustrated in Figure 5e. The overall NORR process on Fe_3_C/Fe_3_N@C involves several synergistic steps: efficient NO diffusion to the catalyst surface, robust NO adsorption and activation, rapid *H generation via H_2_O dissociation, and stepwise hydrogenation. The graphitic carbon‐encapsulated Fe_3_C/Fe_3_N Janus nanostructure promotes all these key processes simultaneously, leading to significantly enhanced electrochemical NORR performance.

## 3 Conclusion

In summary, we developed a self‐supported Fe_3_C/Fe_3_N@C catalyst composed of defect‐rich, graphitic‐carbon‐confined Fe_3_C/Fe_3_N Janus nanostructures for efficient electrochemical NO‐to‐NH_3_ conversion. The catalyst achieves a high NH_3_ yield rate of 468.3 µmol h^−1^ cm^−2^ with a Faradaic efficiency of 94.2% at −0.6 V versus RHE, demonstrating the effectiveness of this multifunctional architecture. Beyond the performance improvement, this study shows that the key kinetic requirements of NORR can be addressed within a single catalyst design. The graphitic carbon shell promotes NO transport across the interfacial water environment, while the Fe_3_C/Fe_3_N Janus interface provides complementary active sites for NO activation and H_2_O dissociation. In this architecture, Fe_3_C favors NO adsorption and activation, whereas nitrogen‐vacancy‐rich Fe_3_N facilitates *H generation for stepwise hydrogenation toward NH_3_. These results establish carbon‐confined, earth‐abundant carbide/nitride Janus interfaces as a useful platform for coupling mass‐transfer regulation with interfacial reactivity control, offering a practical design principle for advanced NORR catalysts and other gas‐involving electrocatalytic transformations.

## Author Contributions

**Jialing Song**: investigation, writing – original draft, methodology, visualization, formal analysis. **Ziqi Wei**: investigation, writing – review and editing, methodology. **Haotian Huang**: investigation, visualization, writing – review and editing. **Lupeng Han**: methodology, formal analysis, writing – review and editing. **Biyi Huang**: methodology, writing – review and editing. **Evangelina Pensa**: writing – review and editing, data curation, formal analysis. **Sam Fong Yau Li**: funding acquisition, writing – review and editing, formal analysis, data curation, resources, supervision. **Eslam Hamed**: formal analysis, writing – review and editing, investigation, validation, data curation. **Fun Man Fung**: writing – review and editing, validation, data curation. **Emiliano Cortés**: writing – review and editing, funding acquisition, conceptualization, supervision, resources, validation. **Dengsong Zhang**: conceptualization, investigation, writing– review and editing, funding acquisition, supervision, resources, project administration.

## Conflicts of Interest

The authors declare no conflicts of interest.

## Supporting information



The detailed experimental section and additional figures and tables are listed in the Supporting Information file. The authors have cited additional references within the Supporting Information.**Supporting File**: anie73787‐sup‐0001‐SuppMat.docx.

## Data Availability

The data that support the findings of this study are available from the corresponding author upon reasonable request.
