# Supplementary material for: Carbon‐Confined Fe3C/Fe3N Janus Interfaces for Selective Nitric Oxide‐to‐Ammonia Electroreduction
Source: Angew Chem Int Ed Engl. 2026 Jul 24;65(39):e1030679. doi: 10.1002/anie.1030679 (PMC13592314; doi:10.1002/anie.1030679)
Supplement: Supplementary file 1 [file ANIE-65-e1030679-s001.docx]

***Supporting Information***

**Carbon-Confined Fe_3_C/Fe_3_N Janus Interfaces for Selective Nitric Oxide-to-Ammonia Electroreduction**

Jialing Song,^[a]^ Ziqi Wei,^[a]^ Haotian Huang,^[a]^ Lupeng Han,*^[a]^ Biyi Huang,^[a]^ Evangelina Pensa,^[b]^ Sam Fong Yau Li,^[c]^ Eslam Hamed,^[c]^ Fun Man Fung,^[d]^ Emiliano Cortés,*^[b]^ Dengsong Zhang*^[a]^

[a]	J. Song, Z. Wei, H. Huang, Assoc. Prof. L. Han, B. Huang, Prof. D. Zhang
International Joint Laboratory of Catalytic Chemistry, State Key Laboratory of Materials for Advanced Nuclear Energy, Innovation Institute of Carbon Neutrality, Department of Chemistry, College of Sciences, Shanghai University, Shanghai 200444, China.
E-mail: [lphan@shu.edu.cn](mailto:haiyanduan@shu.edu.cn) and [dszhang@shu.edu.cn](mailto:dszhang@shu.edu.cn)

[b]	Dr. E. Pensa, Prof. E. Cortés
Nanoinstitute Munich, Faculty of Physics, Ludwig-Maximilians-Universität (LMU), Munich 80539, Germany.
E-mail: [Emiliano.Cortes@lmu.de](mailto:Emiliano.Cortes@lmu.de)

[c]	Prof. S. Li, E. M. Hamed
National University of Singapore, Department of Chemistry, 3 Science Drive 3, Singapore 117543, Singapore.

[d]	Prof. F. M. Fung
School of Chemistry, University College Dublin, D04 N2E5 Dublin, Ireland.

**Table of Contents**

**Section A. Experimental Section**

1. Synthesis Procedures

2. Materials characterization.

3. Electrochemical measurements.

4. ^15^N isotope labelling experiment.

5. Determination of NH_3_.

6. In situ gas (NO) adsorption breakthrough measurements.

7. In situ ATR-IRAS spectroscopy.

8. Online DEMS measurements.

9. Electron Paramagnetic Resonance measurement.

10. Calculations of faradaic efficiency (FE) and yield rate.

11. Calculation of the turnover frequency

12. The NORR performance of Fe_3_C/Fe_3_N@C in Zn-NO battery.

13. DFT calculations.

**Section B. Supporting Figures**

Figure S1. SEM image of the self-supported membrane prepared by electrospinning.

Figure S2. HR-TEM image of Fe_3_C/Fe_3_N@C after calcination.

Figure S3. EDS mapping images of Fe_3_C/Fe_3_N@C.

Figure S4. XRD patterns of Fe_3_N@C.

Figure S5. HR-TEM image and lattice distance profiles of Fe_3_N@C.

Figure S6. XRD patterns of Fe_3_C@C.

Figure S7. HR-TEM image and lattice distance profiles of Fe_3_C@C.

Figure S8. N_2_ adsorption/desorption isotherms of Fe_3_N@C and Fe_3_C/Fe_3_N@C.

Figure S9. C 1s XPS spectra of Fe_3_N@C and Fe_3_C/Fe_3_N@C.

Figure S10. Proposed atomic structure during the pyrolysis process at 500 ℃.

Figure S11. Fe 2p XPS spectra of Fe_3_N@C and Fe_3_C/Fe_3_N@C.

Figure S12. FT-EXAFS fitting curves of Fe_3_C/Fe_3_N@C, Fe_3_C@C, and Fe_3_N@C.

Figure S13. Experimental curves of EXAFS spectra in *k*-space of Fe_3_C/Fe_3_N@C, Fe_3_N@C, Fe_3_C@C, FeO, Fe_2_O_3_, and Fe foil.

Figure S14. FT-EXAFS fitting curves of Fe foil, FeO, and Fe_2_O_3_.

Figure S15. Experimental and fitting curves of EXAFS spectra in *k*-space of Fe_3_C/Fe_3_N@C, Fe_3_N@C, Fe_3_C@C, Fe foil, FeO, and Fe_2_O_3_.

Figure S16. Wavelet transforms EXAFS contour plots of Fe foil.

Figure S17. Wavelet transforms EXAFS contour plots of FeO.

Figure S18. Wavelet transforms EXAFS contour plots of Fe_2_O_3._

Figure S19. (a) UV-Vis absorbance of standard NH_4_^+^ solutions. (b) NH_4_^+^ calibration curve and corresponding fitting queation.

Figure S20. The quantification of hydrazine hydrate (N_2_H_4_). (a) UV-Vis absorbance of standard N_2_H_2_ solutions. (b) N_2_H_2_ calibration curve and corresponding fitting queation.

Figure S21. UV-vis absorption spectra of N_2_H_4_ for Fe_3_C/Fe_3_N@C.

Figure S22. The quantification of hydroxylamine (NH_2_OH). (a) UV-Vis absorbance of standard NH_2_OH solutions. (b) NH_2_OH calibration curve and corresponding fitting queation.

Figure S23. UV-vis absorption spectra of NH_2_OH for Fe_3_C/Fe_3_N@C.

Figure S24. DEMS signals of by-products during NORR.

Figure S25. DEMS signals of NH_3_ and H_2_ over Fe_3_C/Fe_3_N@C, Fe_3_C@C, and Fe_3_N@C during NORR.

Figure S26. Product distributions for Fe_3_C/Fe_3_N@C, Fe_3_C@C, and Fe_3_N@C at -0.6 V vs RHE.

Figure S27. (a) ^1^H NMR spectra of standard NH_4_^+^ solutions. C_4_H_4_O_4_ with a constant concentration was used as an external standard (with a proton signal at d = 6.25 ppm). (b) Calibration curve for ^14^NH_4_^+^ detection using ^1^H NMR, where ^14^NH_4_^+^ peak area integrals were normalized to that of C_4_H_4_O_4_.

Figure S28. The comparison of NH_3_ yield rate and FE using IBS and NMR method.

Figure S29. NORR performance of Fe_3_C/Fe_3_N@C under series conditions (O/C and NFs represent open circuit potential and carbon nanofibers).

Figure S30. CV curves of (a) Fe_3_C/Fe_3_N@C, (b) Fe_3_C@C, and (c) Fe_3_N@C in non-Faradaic area (scan rate: 20 mV s^-1^ to 120 mV s^-1^).

Figure S31. TOF plots for Fe_3_C/Fe_3_N@C, Fe_3_N@C and Fe_3_C@C catalysts.

Figure S32. The Nyquist plots·of·Fe_3_C/Fe_3_N@C, Fe_3_N@C and Fe_3_C@C.

Figure S33. I-t curve at -0.6 V vs RHE for the Fe_3_C/Fe_3_N@C.

Figure S34. HR-TEM image and lattice distance profiles of Fe_3_C/Fe_3_N@C after NORR.

Figure S35. XRD patterns of Fe_3_C/Fe_3_N@C before and after NORR.

Figure S36. C 1s XPS spectra of Fe_3_C/Fe_3_N@C before and after NORR process.

Figure S37. N 1s XPS spectra of Fe_3_C/Fe_3_N@C before and after NORR process.

Figure S38. Fe 2p XPS spectra of Fe_3_C/Fe_3_N@C before and after NORR process.

Figure S39. Zn-NO battery. (a) Open circuit voltage of Fe_3_C/Fe_3_N@C based Zn-NO battery, (b) Polarization curves and power density of assembled Zn-NO battery, (c) Discharge curves under different current densities, (d) The corresponding NH_3_ yield rate under different current densities, (e) Comparison of the NH_3_ yield rate with other reported Zn-NO batteries.

Figure S40. N-end adsorption energy of NO on Fe_3_C, Fe_3_N, and Fe_3_N-V_N_.

Figure S41. N-end and O-end adsorption energy of NO on Fe_3_C.

Figure S42. Models in the COHP analysis of Co-N bond during NO adsorption on Fe_3_C, Fe_3_N, and Fe_3_N-V_N_ site over Fe_3_C/Fe_3_N@C.

Figure S43. Models in the initial state, transition state and final state of water dissociation on Pristine Fe_3_C/Fe_3_N@C.

Figure S44. Models in the initial state, transition state and final state of water dissociation on Fe_3_C/Fe_3_N@C.

Figure S45. Energy barriers during the process of water dissociation about (a) Pristine Fe_3_C/Fe_3_N@C and (b) Fe_3_C/Fe_3_N@C.

Figure S46. Gaussian fits on O-H stretching bands of Fe_3_C@C.

Figure S47. The relative proportions of three types of water about Fe_3_C@C.

Figure S48. Gaussian fits on O-H stretching bands of Fe_3_N@C.

Figure S49. The relative proportions of three types of water about Fe_3_N@C.

Figure S50. Gaussian fits on O-H stretching bands of Fe_3_C/Fe_3_N@C.

Figure S51. In situ ATR-IRAS measurements during 60-min test (at -0.6 V vs. RHE).

Figure S52. DEMS signals of NO during NORR.

Figure S53. Models of reaction pathway on Fe_3_C/Fe_3_N@C.

Figure S54. Models of reaction pathway on Fe_3_N@C.

Figure S55. Models of reaction pathway on Fe_3_C@C.

Figure S56. Gibbs free energy diagrams of NO reduction to NH_3_ over the Fe_3_C/Fe_3_N@C.

Figure S57. Models of reaction pathway on Pristine Fe_3_C/Fe_3_N@C.

Figure S58. Gibbs free energy diagrams of NO reduction to NH_3_ over the Pristine Fe_3_C/Fe_3_N@C.

**Section C. Supporting Tables**

Table S1. Refined atomic positions, occupancies, and isotropic displacement parameters from XRD Rietveld refinement of Fe_3_C/Fe_3_N@C.

Table S2. XRD Rietveld Refinement Results of Fe_3_C/Fe_3_N@C (Antanase).

Table S3. Different percentages of N content in N 1s spectra of Fe_3_N@C and Fe_3_C/Fe_3_N@C.

Table S4. Contents of species in the samples quantified by element analyzer (EA) measurements.

Table S5. BET analysis of as-prepared electrodes materials.

Table S6. EXAFS fitting parameters at the Fe K-edge for various samples

Table S7. Comparison of NORR performances over Fe_3_C/Fe_3_N@C with some previously reported electrocatalysts.

Table S8. Comparison of peak power density with reported battery systems.

## Section A. Experimental Section

## 1.1.Synthesis Procedures

**Synthesis of** **Fe_3_C/Fe_3_N@C.**

The Fe/PAN nanofibers were prepared by utilizing electrospinning technology. Typically, 1.0 g of PAN nanofibers and a calculated amount of Fe(acac)_3_ were dissolved in 10 mL of a mixed solvent solution (DMF : acetone = 8 : 2). After being stirred for 12 h at 60 ℃, the well-mixed solution was transferred to a 10-mL syringe, which was carefully installed onto the electrospinning machine. Subsequently, a continuous and uniform nanofiber sheet was fabricated via electrospinning at a flow rate of 1 mL h⁻¹ under a high voltage (15 kV). The collected nanofiber sheet was peeled off from the aluminum foil, sandwiched between two pieces of translucent tracing paper, and laminated through a preheated laminator (pressure 80-110 micras, temperature 85-145 ℃) to form a stable nanofiber sheet with satisfactory mechanical strength. Subsequently, the nanofiber sheet was pyrolyzed and converted into a carbon fiber sheet in a tube furnace. Firstly, the nanofiber sheet was pre-oxidized at 280 ℃ for 2 h under air atmosphere, and then carbonized at 900 ℃ for 2 hours under a N_2_ gas flow. Next, the sample was naturally cooled to room temperature in a N_2_ atmosphere. Finally, a rectangular piece (1.0 cm×1.0 cm) of the carbonized nanofiber sheet was cut, and Fe_3_C/Fe_3_N@C was obtained.

**Synthesis of Fe_3_N@C.**

The preparation process of Fe_3_N@C is similar to that of Fe_3_C/Fe_3_N@C, except that the calcination temperature in N_2_ atmosphere is 500 ℃.

**Synthesis of Fe_3_C@C.**

The preparation process of Fe_3_C@C is similar to that of Fe_3_C/Fe_3_N@C, except that the polymer is polyvinylidene difluoride (PVDF).

### 1.2.Materials characterization.

The morphologies of the samples were observed by scanning electron microscopy (SEM, Sigma-300) and transmission electron microscopy (TEM, Hitachi-7700) under accelerating voltages of 20 kV and 300 kV respectively. Energy dispersive X-ray spectroscopy (EDX) analysis was carried out by the TEM instrument (JEM-2100F). The composition of the catalyst was analyzed by Agilent 8900 inductively coupled plasma mass spectrometry (ICP-MS). X-ray diffraction (XRD, Bruker, D8-Advance) was performed using a diffractometer with Cu Kα radiation (λ = 1.5406 Å) at a scanning rate of 5° min^-1^ to determine the crystal phase of the catalyst. X-ray photoelectron spectroscopy (XPS) was performed on a PHI 5000 Versaprobe system using monochromatic Al Kα radiation. All binding energies were referenced to the C 1s peak at 284.8 eV. The Brunauer-Emmett-Teller surface area was measured through nitrogen absorption-desorption isotherm at 77 K on a nitrogen absorption apparatus (Quadrasorb-evo, USA). The ultraviolet-visible (UV-Vis) absorbance spectra were measured on a Cary5000 UV-Vis spectrophotometer (Agilent, USA). The Nuclear magnetic resonance (NMR) measurement was carried out on an AVANCE NEO 600M NMR spectrometer (Bruker, Germany). Fourier transform infrared spectroscopy (FTIR) was performed with a Nicolet 6700 instrument equipped with an MCT detector. Differential electrochemical mass spectrometry (DEMS, QAS 100) was performed by Linglu Instruments (Shanghai) Co., Ltd., to perform online analysis of the produced intermediates and products. The Fe K-edge X-ray absorption fine structure (XAFS) were performed in fluorescence excitation mode at the BL14W1 beamline of the Shanghai Synchrotron Radiation Facility. The EXAFS raw data were calibrated with standard Fe foil. Then, XANES and EXAFS data were processed with the Athena, Artemis, and IFEFFIT software packages.

### 1.3.Electrochemical measurements.

Electrochemical measurements were performed in a sealed H-type electrochemical cell separated by a piece of Nafion 117 membrane with a three-electrode system at the room temperature (electrochemical workstation, CHI 660E, CH Instruments Ins, Shanghai). The membrane was treated firstly in H_2_O_2_ (5 wt%) aqueous solution at 80 °C for 1 h. And then, it treated in 0.5 M H_2_SO_4_ for 2 h at 80 °C and finally in water for 6 h. The catalyst was cut into rectangles of 1 cm × 1 cm and directly used as a working electrode and Ag/AgCl (saturated KCl electrolyte) was used as the reference electrode. The potentials reported in this work were converted to RHE scale via calibration with the following equation: E (vs. RHE) = E (vs. Ag/AgCl) + 0.197 + 0.059 × pH, and the polarization curves were the steady-state ones after several cycles. The electrochemical activity of electrocatalysts was measured for NORR. Before electrochemical tests, the electrolyte was firstly purged with high purity Ar gas (99.999%) for 30 min to exclude the air. And the NO gas (10%) was then fed for 30 min to saturate the electrolyte and kept constant during the electrochemical tests.

### 1.4.^15^N isotope labelling experiment.

The ^15^N isotope labelling experiment was proceeded on the ^1^H NMR spectroscopy (600 MHz) to identify the nitrogen source for NORR. A gas mixture of 10 vol% ^15^NO (99% purity) in Ar was used for isotope labeling experiments. Before electrochemical tests, the electrolyte was firstly purged with high purity Ar gas (99.999%) for 30 min to exclude the air. And the ^15^NO gas was then fed for 30 min to saturate the electrolyte and kept constant during the electrochemical tests. The reaction was carried out at -0.6 V vs RHE for 1 h in 0.5 M Na_2_SO_4_. In a typical procedure, 2 mL of the catholyte after reaction was collected and acidized by H_2_SO_4_ (1mL, 1M). After that, the neutralized electrolyte (60 μL), maleic acid (as the internal standard) aqueous solution (20 μL, 3.6mM), and DMSO-d_6_ (500 μL) were added into an NMR tube. Then the sealed mixture was analysed by ^1^H NMR measurement.

### 1.5.Determination of NH_3_.

The yield of the NH_3_ was determined by a spectrophotometry measurement with the indophenol blue method and NMR. For indophenol blue method, a certain amount of electrolyte was taken out from the electrolytic cell and diluted to 2 mL to the detection range. Then, 2 mL of a 1 M NaOH solution that contained salicylic acid and sodium citrate was added. Then, 1 mL of 0.05 M NaClO and 0.2 mL of 1 wt% C_5_FeN_6_Na_2_O·2H_2_O were added to the above solution. After standing at room temperature for 2 h, the UV-Vis absorption spectrum was measured. The concentration of NH_3_ was determined using the absorbance at a wavelength of 655 nm. The concentration-absorbance curve was calibrated using a series of standard NH_4_Cl solutions. The fitting curve (y = 0.4157x − 0.0107, R^2^ = 0.999) showed good linear relation of absorbance value with NH_3_ concentrations. For NMR, After the NO electroreduction, the electrolyte was taken out, and the pH value was adjusted to be weak acid with 0.5 M HCl for further quantification by ^1^H NMR (600 MHz) with external standards of dimethyl sulfoxide (DMSO, 5.16 mM), number of scans is 256 in all NMR tests. The fitting curve (y =2.7356x – 1.7665, R^2^ = 0.9994) shows a good linear relation.

### 1.6.In situ gas (NO) adsorption breakthrough measurements.

In situ gas (NO) adsorption breakthrough tests combined with mass spectrometry (MS) were carried out using the dynamic sorption analyzer (mixSorb S) with a thermal conductivity detector (TCD) combined with a mass spectrometer (OMNISTARTM).

### 1.7.In situ ATR-IRAS spectroscopy.

ATR-IRAS experiments were conducted with a Bruker INVENIO R spectrometer equipped with a liquid nitrogen-cooled mercury cadmium telluride A (MCT-A) detector and the spectro-electrochemical cell was custom-made by Ling Lu Instruments, Shanghai. To improve the signal intensity, a layer of gold was deposited on the surface of monocrystalline silicon by electroless plating. The ink from the sample was dropped on the surface of the Au film and served as the working electrode. Platinum foil and Ag/AgCl electrodes were used as the counter and reference electrodes, respectively. Potential-dependent in situ ATR-IRAS was carried out with an i-t test from -0.3 V to -0.9 V (vs RHE) with a scan rate of 10 mV s^-1^.

### 1.8.Online DEMS measurements.

The DEMS measurements were recorded by QAS 100 spectrometer (Ling Lu Instruments, Shanghai). The 0.5M Na_2_SO_4_ electrolyte was first purged with high-purity Ar (99.999%) for 30 min to remove air. Then, 10% NO was bubbled into the electrolyte to saturation and kept constant during the DEMS measurements. As-prepared single-atom electrocatalysts, platinum wire, and Ag/AgCl electrode were used as the working, counter, and reference electrodes, respectively. A potentiostatic test at -0.6 V vs RHE was performed, and the corresponding mass signals could be detected during this period. After the electrochemical test, the mass signal returned to baseline, and the next cycle was started under the same test conditions. The measurement ended after four consecutive cycles.

### 1.9.Electron Paramagnetic Resonance measurement.

The EPR tests was carried out on the EPR200-PLUS model equipment (China Guoyi Quantum). The test temperature was 298 K, the modulation frequency is 100 kHz, the microwave power is 1 mW, the microwave frequency is 9.52 GHz, the scanning center is 3398 G, the scanning width is 100 G, and the modulation amplitude is 1 G.

### 1.10. Calculations of faradaic efficiency (FE) and yield rate.

The FE for NH_3_ electrosynthesis was defined as the amount of electric charge used for producing NH_3_ divided by the total charge passed through the electrodes during the electrolysis. The FE was calculated according to the following equation:

FE = n × F × c × V / (M × Q) (1)

The NH_3_ yield rate was calculated using the following equation:

NH_3_ yield rate = c_NH3_ × V / (17 × t × S) (2)

Where n is the number of electrons was needed to produce one product molecule; F is Faraday constant (96485 C mol^-1^); c is the measured mass concentration of product; V is the volume of the cathodic reaction electrolyte; M is relative molecular mass of specific product; Q is the quantity of applied charge/electricity; t is the time for which the potential was applied; S is the geometric area of the working electrode (1 cm^2^).

### 1.11. Calculation of the turnover frequency

The TOF value was calculated based on the following formula:

$$TOF=\frac{j\times A}{n\times N_{active}\times F}$$

Where:

j: current density (A cm^-2^) at a specific overpotential;

A: geometric electrode area (cm^2^);

n: number of electron transfer per product molecule (the value of n is 5 for NORR);

F: Faraday constant (96485 C mol^-1^);

N_active_: number of moles of active sites (mol).

The number of active sites was determined from the electrochemical surface area (ECSA) and surface atom density:

$$N_{active}=\frac{ECSA\times\rho_{surface}}{N_{A}}$$

Where ECSA is the electrochemical surface area (cm^2^), obtained from double-layer capacitance measurements. $\rho_{surface}$ is the surface atom density (atoms cm^-2^), determined by the unit cell method. N_A_ is the Avogadro's constant (6.022 × 10^23^ mol^-1^). To estimate the number of surface atoms per ECSA, the crystallographic parameters of Fe_3_C and Fe_3_N are taken into account. Based on the unit cell volumes (155.42 Å^3^ for Fe_3_C and 98.49 Å^3^ for Fe_3_N) and the atomic arrangements on the respective facets, the surface atom densities were calculated to be 5.18 × 10^14^ atoms·cm^-2^ for the Fe_3_C (031) facet and 5.41 × 10^14^ atoms·cm^-2^ for the Fe_3_N (111) facet. For Fe_3_C/Fe_3_N, considering the interfacial contribution, the number of surface atoms per ECSA is estimated to be 5.30 × 10^14^ atoms·cm^-2^. By substituting the obtained terms in formula, the TOF can be calculated.

### 1.12.The NORR performance of Fe_3_C/Fe_3_N@C in Zn-NO battery.

The Zn-NO battery was constructed using an H-cell. The self-supported Fe_3_C/Fe_3_N@C electrode was employed as the cathode to perform the NORR in a cathodic electrolyte (0.5 M Na_2_SO_4_), respectively. A polished Zn plate was set in an anodic electrolyte (0.5 M NaOH), and a Nafion 117 membrane was used to separate the two different electrolytes. During the battery discharge process, the Zn-NO electrochemistry implements electrochemical NO reduction driven by Zn dissolution.

### 1.13.DFT calculations.

Considering the limitations of computational power, we opted for a simplified model, namely a carbon-encapsulated Fe_3_C/Fe_3_N Janus heterostructure, combining the experimentally observed morphology and the intimate interface between Fe_3_C and Fe_3_N. The choice of using crystal facets of Fe_3_C (031) and Fe_3_N (111) to construct the model is primarily based on HRTEM and XRD characterizations. These crystal facets exhibit the strongest diffraction peaks in the XRD patterns and are also among the thermodynamically stable facets. It is noted that the Fe_3_C (031) and Fe_3_N (111) slabs are taken from the optimized bulk structures of Fe_3_C and Fe_3_N, respectively. Based on the strong EPR and XPS evidence, we introduced nitrogen vacancies into the Fe_3_N. To mimic the graphitic carbon‑encapsulated structure, we added graphene layers below the Fe_3_C/Fe_3_N slab, which provides a realistic electronic environment for the encapsulated active interface.

The spin-polarized density functional theory (DFT) calculations were carried out using the Vienna ab initio simulation package (VASP) based on the plane-wave basis sets with the projector augmented-wave method. The exchange-correlation potential was treated by employing a generalized gradient approximation (GGA) with the Perdew-Burke-Ernzerhof (PBE) parametrization. The energy cutoff was set at 400 eV. The Brillouin-zone integration was sampled with a Γ-centered Monkhorst-Pack mesh of 1×1×1 by VASPKIT. The structures were fully relaxed until the energy convergence criterion was 10^-5^ eV. The van der Waals correction of Grimme's DFT-D3 model was also adopted. To avoid periodic interactions for interface structures, a vacuum layer as large as 10 Å is used along the c direction normal to the interface. The adsorption energy (E_ads_) of NO is calculated according to the following equation:

E_ads_ = E_total_ - E_NO_ - E_surface_

where E_total_, E_NO_, and E_surface_ represent the total energy of the system, the energy of the isolated NO molecule, and the energy of the surface, respectively. The bigger absolute value of the E_ads_ indicates a stronger adsorption strength in the system.

The binding energy (ΔE_H_) of hydrogen is calculated according to the following equation:

ΔE_H_ = E_total_ - (E_H2_)/2 - E_surface_

where E_total_, E_H2_, and E_surface_ represent the total energy of the system, the energy of the isolated H_2_ molecule, and the energy of the surface, respectively.

“ΔG_H_” was used to calculate ΔG_H_ = ΔE_H_ + 0.24 eV, which is taken for the following calculations.

For each subsequent elementary step, the Gibbs free energy is calculated after gas correction by：

Δ*G* = Δ*E* − ΔZPE + *T*Δ*S*

where ΔE is the reaction energy obtained by the total energy difference between the reactant and product molecules absorbed on the catalyst surface and Δ*S* is the change in entropy for each reaction. The room temperature *T* = 298.15 K is used in the calculations to take temperature influence into consideration. ΔZPE is the zero-point energy correction to the Gibbs free energy.

## Section S2. Supplementary characterizations.

### 2.1. Supplemental Figures

**Figure S1.** SEM image of the self-supported membrane prepared by electrospinning.

**Figure S2.** HR-TEM image of Fe_3_C/Fe_3_N@C after calcination.

**Figure S3.** EDS mapping images of Fe_3_C/Fe_3_N@C.

**Figure S4.** XRD patterns of Fe_3_N@C.

**Figure S5**. HR-TEM image and lattice distance profiles of Fe_3_N@C.

**Figure S6.** XRD patterns of Fe_3_C@C.

**Figure S7.** HR-TEM image and lattice distance profiles of Fe_3_C@C.

**Figure S8.** N_2_ adsorption/desorption isotherms of Fe_3_N@C and Fe_3_C/Fe_3_N@C.

**Figure S9.** C 1s XPS spectra of Fe_3_N@C and Fe_3_C/Fe_3_N@C.

**Figure S10.** Proposed atomic structure during the pyrolysis process at 500 ℃.

**Figure S11.** Fe 2p XPS spectra of Fe_3_N@C and Fe_3_C/Fe_3_N@C.

**Figure S12.** FT-EXAFS fitting curves of Fe_3_C/Fe_3_N@C, Fe_3_C@C, and Fe_3_N@C.

**Figure S13.** Experimental curves of EXAFS spectra in *k*-space of Fe_3_C/Fe_3_N@C, Fe_3_N@C, Fe_3_C@C, FeO, Fe_2_O_3_, and Fe foil.

**Figure S14.** FT-EXAFS fitting curves of Fe foil, FeO, and Fe_2_O_3_.

**Figure S15.** Experimental and fitting curves of EXAFS spectra in *k*-space of Fe_3_C/Fe_3_N@C, Fe_3_N@C, Fe_3_C@C, Fe foil, FeO, and Fe_2_O_3_.

**Figure S16.** Wavelet transforms EXAFS contour plots of Fe foil.

**Figure S17.** Wavelet transforms EXAFS contour plots of FeO.

**Figure S18.** Wavelet transforms EXAFS contour plots of Fe_2_O_3_.

**Figure S19.** (a) UV-Vis absorbance of standard NH_4_^+^ solutions. (b) NH_4_^+^ calibration curve and corresponding fitting equation.

**Figure S20.** The quantification of hydrazine hydrate (N_2_H_4_). (a) UV-Vis absorbance of standard N_2_H_2_ solutions. (b) N_2_H_2_ calibration curve and corresponding fitting equation.

**Figure S21.** UV-vis absorption spectra of N_2_H_4_ for Fe_3_C/Fe_3_N@C.

**Figure S22.** The quantification of hydroxylamine (NH_2_OH). (a) UV-Vis absorbance of standard NH_2_OH solutions. (b) NH_2_OH calibration curve and corresponding fitting equation.

**Figure S23.** UV-vis absorption spectra of NH_2_OH for Fe_3_C/Fe_3_N@C.

**Figure S24**. DEMS signals of by-products during NORR.

**Figure S25**. DEMS signals of NH_3_ and H_2_ over Fe_3_C/Fe_3_N@C, Fe_3_C@C, and Fe_3_N@C during NORR.

**Figure S26.** Product distributions for Fe_3_C/Fe_3_N@C, Fe_3_C@C, and Fe_3_N@C at -0.6 V vs RHE.

**Figure S27.** (a) ^1^H NMR spectra of standard NH_4_^+^ solutions. C_4_H_4_O_4_ with a constant concentration was used as an external standard (with a proton signal at d = 6.25 ppm). (b) Calibration curve for ^14^NH_4_^+^ detection using ^1^H NMR, where ^14^NH_4_^+^ peak area integrals were normalized to that of C_4_H_4_O_4_.

**Figure S28.** The comparison of NH_3_ yield rate and FE using IBS and NMR method.

**Figure S29.** NORR performance of Fe_3_C/Fe_3_N@C under series conditions (O/C and NFs represent open circuit potential and carbon nanofibers).

**Figure S30.** CV curves of (a) Fe_3_C/Fe_3_N@C, (b) Fe_3_C@C, and (c) Fe_3_N@C in non-Faradaic area (scan rate: 20 mV s^-1^ to 120 mV s^-1^).

**Figure S31.** TOF plots for Fe_3_C/Fe_3_N@C, Fe_3_N@C and Fe_3_C@C catalysts.

**Figure S32.** The Nyquist plots·of·Fe_3_C/Fe_3_N@C, Fe_3_N@C and Fe_3_C@C. (R_s_ represents the uncompensated solution resistance between the working electrode and the reference electrode. CPE is the constant phase element used to model the double-layer capacitance. R_ct_ is the charge-transfer resistance associated with the Faradaic process of the NO reduction reaction.)

**Figure S33.** I-t curve at -0.6 V vs RHE for the Fe_3_C/Fe_3_N@C (Replace the electrolyte every 10 hours).

**Figure S34**. HR-TEM image and lattice distance profiles of Fe_3_C/Fe_3_N@C after NORR.

**Figure S35.** XRD patterns of Fe_3_C/Fe_3_N@C before and after NORR.

**Figure S36.** C 1s XPS spectra of Fe_3_C/Fe_3_N@C before and after NORR process.

**Figure S37.** N 1s XPS spectra of Fe_3_C/Fe_3_N@C before and after NORR process.

**Figure S38.** Fe 2p XPS spectra of Fe_3_C/Fe_3_N@C before and after NORR process.

**Figure S39.** Zn-NO battery. (a) Open circuit voltage of Fe_3_C/Fe_3_N@C based Zn-NO battery, (b) Polarization curves and power density of assembled Zn-NO battery, (c) Discharge curves under different current densities, (d) The corresponding NH_3_ yield rate under different current densities, (e) Comparison of the NH_3_ yield rate with other reported Zn-NO batteries.

As an environmentally sustainable energy device, the Zn-NO battery holds the remarkable ability to concurrently achieve the removal of nitric oxide, the generation of ammonia, and the output of electrical energy. In this study, we constructed a proof-of-concept Zn-NO battery. The cathode of this battery is composed of a self-supported Fe_3_C/Fe_3_N@C catalyst, while the anode is a Zn plate. As clearly demonstrated in Figure S39a, this battery prototype can deliver a high open-circuit voltage of 1.99 V vs Zn. Notably, this value is close to the theoretical value of 1.87 V vs Zn and is in good agreement with the polarization curve presented in Figure S39b. What's more, the maximum power density of this battery is calculated to reach an impressive 16.28 mW cm^-2^, indicating its great potential for practical applications. Figure S39c shows the discharge curves under different current densities ranging from 0.5 to 8 mA cm^-2^. Under each tested current density, the output voltage remained relatively stable for 1 h, which vividly demonstrates the good discharge performance of the battery. Additionally, after discharging under each current density, we collected the electrolyte and measured the corresponding NH_3_ yield rate under different current densities (Figure S39d). Under the condition of 8 mA cm^-2^, the NH_3_ yield rate reached a maximum value of 519.8 μg h^-1^ cm^-2^. Overall, the self-supported Fe_3_C/Fe_3_N@C based Zn-NO battery we assembled showcases excellent performance, featuring high power density and NH_3_ yield rate. This performance is superior to that of most Zn-NO batteries recently reported (Figure S39e and Table S8).

**Figure S40.** N-end adsorption energy of NO on Fe_3_C, Fe_3_N, and Fe_3_N-V_N_.

**Figure S41.** N-end and O-end adsorption energy of NO on Fe_3_C.

**Figure S42.** Models in the COHP analysis of Co-N bond during NO adsorption on Fe_3_C, Fe_3_N, and Fe_3_N-V_N_ site over Fe_3_C/Fe_3_N@C.

**Figure S43**. Models in the initial state, transition state and final state of water dissociation on Pristine Fe_3_C/Fe_3_N@C.

**Figure S44.** Models in the initial state, transition state and final state of water dissociation on Fe_3_C/Fe_3_N@C.

**Figure S45.** Energy barriers during the process of water dissociation about (a) Pristine Fe_3_C/Fe_3_N@C and (b) Fe_3_C/Fe_3_N@C.

**Figure S46.** Gaussian fits on O-H stretching bands of Fe_3_C@C.

**Figure S47.** The relative proportions of three types of water about Fe_3_C@C.

**Figure S48.** Gaussian fits on O-H stretching bands of Fe_3_N@C.

**Figure S49.** The relative proportions of three types of water about Fe_3_N@C.

**Figure S50.** Gaussian fits on O-H stretching bands of Fe_3_C/Fe_3_N@C.

**Figure S51**. In situ ATR-IRAS measurements during 60-min test (at -0.6 V vs. RHE).

**Figure S52.** DEMS signals of NO during NORR.

**Figure S53.** Models of reaction pathway on Fe_3_C/Fe_3_N@C.

**Figure S54.** Models of reaction pathway on Fe_3_N@C.

**Figure S55.** Models of reaction pathway on Fe_3_C@C.

**Figure S56.** Gibbs free energy diagrams of NO reduction to NH_3_ over the Fe_3_C/Fe_3_N@C.

**Figure S57.** Models of reaction pathway on Pristine Fe_3_C/Fe_3_N@C.

**Figure S58.** Gibbs free energy diagrams of NO reduction to NH_3_ over the Pristine Fe_3_C/Fe_3_N@C.

### 2.2.Supplemental Tables

**Table S1.** Refined atomic positions, occupancies, and isotropic displacement parameters from XRD Rietveld refinement of Fe_3_C/Fe_3_N@C.

| Site | Np | x | y | z | Atom | Occ | Beq |
| --- | --- | --- | --- | --- | --- | --- | --- |
| Fe_1 | 8 | 0.19049 | 0.07387 | 0.34986 | Fe | 1 | 0.428 |
| Fe_2 | 4 | 0.01878 | 0.25000 | 0.80746 | Fe | 1 | 1 |
| C_1 | 4 | 0.96948 | 0.25000 | 0.55729 | C | 1 | 0.9 |

Table S2. XRD Rietveld Refinement Results of Fe_3_C/Fe_3_N@C (Antanase).

|  | Use | Value | Error |
| --- | --- | --- | --- |
| Use phase | 1 |  |  |
| R-Bragg |  | 7.556 |  |
| Spacegroup |  | P_n_m_a |  |
| Scale | 1 | 1.30972e-003 |  |
| Cell Mass |  | 718.183 | 0 |
| Cell Volume (Å^3) |  | 155.77614 | 0 |
| Wt% - Rietveld |  | 91.110 | 0 |
| Double-Voigt\|Approach |  |  |  |
| Cry size Lorentzian |  | 30.0 | 0 |
| k: 1 LVol-IB (nm) |  | 19.099 |  |
| k: 0.89 LVol-FWHM (nm) |  | 26.700 |  |
| Crystal Linear Absorption Coeff. (1/cm) |  | 2155.432 | 0 |
| Crystal Density (g/cm^3) |  | 7.656 | 0 |
| Lattice parameters |  |  |  |
| a (Å) |  | 5.0971916 |  |
| b (Å) |  | 6.7461928 |  |
| c (Å) |  | 4.5301360 |  |

**Table S3.** Different percentages of N content in N 1s spectra of Fe_3_N@C and Fe_3_C/Fe_3_N@C.

| Samples | Pyridinic N (%) | Fe-N | Pyrrolic N (%) | Graphitic N (%) | Oxidized N (%) |
| --- | --- | --- | --- | --- | --- |
| Fe_3_N@C | 4.14 | 3.82 | 3.69 | 0.89 | 0.93 |
| Fe_3_C/Fe_3_N@C | 1.46 | 1.09 | 1.66 | 1.34 | 0.42 |

**Table S4.** Contents of species in the samples quantified by element analyzer (EA) measurements.

| Samples | Fe_3_N@C | Fe_3_C/Fe_3_N@C |
| --- | --- | --- |
| Content of N (wt.%) | 8.02 | 1.59 |
| Content of C (wt.%) | 63.27 | 76.64 |

**Table S5.** BET analysis of as-prepared electrodes materials.

| Parameters | Fe_3_N@C | Fe_3_C@C | Fe_3_C/Fe_3_N@C |
| --- | --- | --- | --- |
| BET surface area (m^2^ g^-1^) | 109.61 | 233.08 | 312.95 |
| Total pore volume (cm^3^ g^-1^) | 0.11 | 0.18 | 0.28 |
| Average pore size (nm) | 4.69 | 4.82 | 5.12 |

**Table S6.** EXAFS fitting parameters at the Fe K-edge for various samples.

| Sample | Shell | CN^a^ | R(Å)^b^ | σ^2^(Å^2^)^c^ | ΔE_0_(eV)^d^ | R factor |
| --- | --- | --- | --- | --- | --- | --- |
| Fe foil | Fe-Fe | 8.0* | 2.47±0.01 | 0.0048 | 7.6±1.1 | 0.0018 |
|  | Fe-Fe | 6.0* | 2.85±0.01 | 0.0065 |  |  |
| FeO | Fe-O | 6.0±0.1 | 2.15±0.01 | 0.0133 | 7.0±0.9 | 0.0033 |
|  | Fe-Fe | 12.0±0.3 | 3.03±0.01 | 0.0094 | -6.1±0.5 |  |
| Fe_3_C | Fe-Fe | 4.4±0.6 | 2.51±0.01 | 0.010 | 8.2±1.8 | 0.011 |
|  | Fe-Fe | 2.7±0.6 | 2.77±0.01 | 0.0005 |  |  |
| Fe_2_O_3_ | Fe-O | 3.8±0.2 | 1.94±0.01 | 0.0042 | -2.6±1.7 | 0.0032 |
|  | Fe-O | 2.4±0.3 | 2.10±0.01 |  |  |  |
|  | Fe-Fe | 4.8±0.2 | 2.95±0.01 | 0.0060 | -1.2±1.1 |  |
|  | Fe-Fe | 3.7±0.3 | 3.38±0.01 |  |  |  |
|  | Fe-Fe | 4.9±0.3 | 3.69±0.01 |  |  |  |
| Fe_3_C/Fe_3_N@C | Fe-N/C | 3.8±0.3 | 2.01±0.03 | 0.0089 | 5.4±0.8 | 0.0182 |
|  | Fe-Fe | 2.6±0.3 | 3.01±0.01 | 0.0067 | 9.3±0.6 |  |
| Fe_3_N@C | Fe-N | 2.0±0.1 | 1.95±0.02 | 0.0091 | 6.2±0.6 | 0.010 |
|  | Fe-Fe | 10.2±0.3 | 2.54±0.03 | 0.0091 |  |  |
| Fe_3_C@C | Fe-C | 1.4±0.2 | 1.98±0.03 | 0.0038 | 3.8±0.4 | 0.0168 |
|  | Fe-Fe | 7.3±0.3 | 2.59±0.03 | 0.0038 |  |  |

(a)CN, coordination number; (b)R, distance between absorber and backscatter atoms; (c)σ^2^, Debye-Waller factor to account for both thermal and structural disorders; (d)ΔE_0_, inner potential correction; R factor indicates the goodness of the fit. S_0_^2^ was fixed to 0.753, according to the experimental EXAFS fit of Fe foil by fixing CN as the known crystallographic value. A reasonable range of EXAFS fitting parameters: 0.700 < Ѕ_0_^2^< 1.000; CN > 0; σ^2^Å^2^＞0; |ΔE_0_|< 15 eV; R factor < 0.02.

**Table S7.** Comparison of NORR performances over Fe_3_C/Fe_3_N@C with some previously reported electrocatalysts.

| Catalyst | Electrotyte | Potential  (V vs. RHE) | Yield rate  (μmol h^-1^ cm^-1^) | FE (%) | C_NO_ | Ref. |
| --- | --- | --- | --- | --- | --- | --- |
| Fe_3_C/Fe_3_N@C | 0.5 M Na_2_SO_4_ | -0.6 | 468.26 | 94.18 | 10 vol% | This work |
| CoB/Co@C | 0.5 M Na_2_SO_4_ | -0.6 | 315.4 | 70.3 | 10 vol% | ^[1]^ |
| Fe_2_O_3_/CP | 0.1 M Na_2_SO_4_ | -0.4 | 78.1 | 86.7 | 10 vol% | ^[2]^ |
| FeNC | 0.1 M HClO_4_ | -0.2 | 20.2 | 5.1 | 99.9 vol% | ^[3]^ |
| Ni_2_P/CP | 0.1 M HCl | -0.2 | 33.5 | 76.9 | 10 vol% | ^[4]^ |
| a-B_2.6_C@TiO_2_/Ti | 0.2 M Na_2_SO_4_ | -0.9 | 79.4 | 89 | 10 vol% | ^[5]^ |
| MoS_2_/GF | 0.1 M HCl | -0.7 | 99.6 | 76.6 | 10 vol% | ^[6]^ |
| bcc RuGa-2 IMCs | 0.1 M K_2_SO_4_ | -0.2 | 160.3* | 72.3 | 20 vol% | ^[7]^ |
| NiO/TM | 0.1 M Na_2_SO_4_ | -0.6 | 125.3 | 90 | 10 vol% | ^[8]^ |
| FeP/CC | 0.2 M PBS | -0.3 | 85.6 | 61* | 10 vol% | ^[9]^ |
| NiNC@CF | 0.5 M PBS | -0.9 | 108 | 52 | 10 vol% | ^[10]^ |
| BF-COF | 0.5 M Na_2_SO_4_ | -0.6 | 68.62 | 68.85 | 10 vol% | ^[11]^ |
| FeOCl-V_Cl_ | 0.1 M HCl | -0.5 | 455.38 | 91.06 | 10 vol% | ^[12]^ |
| Cu-Co_3_O_4_ NBs | 0.5 M Na_2_SO_4_ | -0.4 | 3.19 | 94.13 | 1 vol% | ^[13]^ |

*The data were calculated according to the curves given in the literature.

**Table S8.** Comparison of peak power density with reported battery systems.

| Catalysts | Battery system | Power density  (mW cm^-2^) | Ref. |
| --- | --- | --- | --- |
| Fe_3_C/Fe_3_N@C | metal-NO | 16.28 | This work |
| hcp-Co | metal-NO | 4.66 | ^[14]^ |
| O_v_-Cu_2_O | metal-NO | 4.62 | ^[15]^ |
| CoB/Co@C | metal-NO | 3.68 | ^[16]^ |
| Cu@Co | metal-NO | 3.08 | ^[17]^ |
| Bi@C | metal-NO | 2.35 | ^[18]^ |
| CuFe DS/NC | metal-NO | 2.30 | ^[19]^ |
| Cu-OUC | metal-NO | 2.04 | ^[20]^ |
| Fe_2_O_3_/CP | metal-NO | 1.18 | ^[2]^ |
| Ni_2_P/CP | metal-NO | 1.53 | ^[4]^ |
| MoS_2_/GF | metal-NO | 1.04 | ^[6]^ |
| NiO/TM | metal-NO | 0.88 | ^[8]^ |
| a-B_2.6_C@TiO_2_/Ti | metal-NO | 1.7 | ^[21]^ |
| CoPi/NPCS | metal-N_2_ | 0.49 | ^[22]^ |
| NbS_2_ | metal-N_2_ | 0.31 | ^[23]^ |

## Reference

[1]	B. C. Wu, L. Huang, L. Yan, H. Y. Gang, Y. Y. Cao, D. Wei, H. Y. Wang, Z. P. Guo, W. C. Zhang, "Boron-Modulated Electronic-Configuration Tuning of Cobalt for Enhanced Nitric Oxide Fixation to Ammonia" *Nano Letters* **2023**, *23*, 7120-7128.

[2]	J. Liang, H. Y. Chen, T. Mou, L. C. Zhang, Y. T. Lin, L. C. Yue, Y. S. Luo, Q. Liu, N. Li, A. A. Alshehri, I. Shakir, P. O. Agboola, Y. Y. Wang, B. Tang, D. W. Ma, X. P. Sun, "Coupling denitrification and ammonia synthesis via selective electrochemical reduction of nitric oxide over Fe_2_O_3_ nanorods" *Journal of Materials Chemistry A* **2022**, *10*, 6454-6462.

[3]	D. H. Kim, S. Ringe, H. Kim, S. Kim, B. Kim, G. Bae, H. S. Oh, F. Jaouen, W. Kim, H. Kim, C. H. Choi, "Selective electrochemical reduction of nitric oxide to hydroxylamine by atomically dispersed iron catalyst" *Nature Communications* **2021**, *12,* 1856.

[4]	T. Mou, J. Liang, Z. Y. Ma, L. C. Zhang, Y. T. Lin, T. S. Li, Q. Liu, Y. L. Luo, Y. Liu, S. Y. Gao, H. T. Zhao, A. M. Asiri, D. W. Ma, X. P. Sun, "High-efficiency electrohydrogenation of nitric oxide to ammonia on a Ni_2_P nanoarray under ambient conditions" *Journal of Materials Chemistry A* **2021**, *9*, 24268-24275.

[5]	G. Meng, M. M. Jin, T. R. Wei, Q. Liu, S. S. Zhang, X. Y. Peng, J. Luo, X. J. Liu, "MoC nanocrystals confined in N-doped carbon nanosheets toward highly selective electrocatalytic nitric oxide reduction to ammonia" *Nano Research* **2022**, *15*, 8890-8896.

[6]	L. C. Zhang, J. Liang, Y. Y. Wang, T. Mou, Y. T. Lin, L. C. Yue, T. S. Li, Q. Liu, Y. L. Luo, N. Li, B. Tang, Y. Liu, S. Y. Gao, A. A. Alshehri, X. D. Guo, D. W. Ma, X. P. Sun, "High-Performance Electrochemical NO Reduction into NH_3_ by MoS_2_ Nanosheet" *Angewandte Chemie-International Edition* **2021**, *60*, 25263-25268.

[7]	H. F. Zhang, Y. B. Li, C. Q. Cheng, J. Zhou, P. F. Yin, H. M. Wu, Z. Q. Liang, J. W. Zhang, Q. B. Yun, A. L. Wang, L. J. Zhu, B. Zhang, W. B. Cao, X. M. Meng, J. Xia, Y. F. Yu, Q. P. Lu, "Isolated Electron-Rich Ruthenium Atoms in Intermetallic Compounds for Boosting Electrochemical Nitric Oxide Reduction to Ammonia" *Angewandte Chemie-International Edition* **2023**, *62,* e202213351.

[8]	P. Y. Liu, J. Liang, J. Q. Wang, L. C. Zhang, J. Li, L. C. Yue, Y. C. Ren, T. S. Li, Y. L. Luo, N. Li, B. Tang, Q. Liu, A. M. Asiri, Q. Q. Kong, X. P. Sun, "High-performance NH_3_ production via NO electroreduction over a NiO nanosheet array" *Chemical Communications* **2021**, *57*, 13562-13565.

[9]	J. Liang, Q. Zhou, T. Mou, H. Y. Chen, L. C. Yue, Y. S. Luo, Q. Liu, M. S. Hamdy, A. A. Alshehri, F. Gong, X. P. Sun, "FeP nanorod array: A high-efficiency catalyst for electroreduction of NO to NH_3_ under ambient conditions" *Nano Research* **2022**, *15*, 4008-4013.

[10]	T. Muthusamy, S. S. Markandaraj, S. Shanmugam, "Nickel nanoparticles wrapped in N-doped carbon nanostructures for efficient electrochemical reduction of NO to NH_3_" *Journal of Materials Chemistry A* **2022**, *10*, 6470-6474.

[11]	D. L. Han, Z. L. Wang, H. Y. Duan, L. P. Yang, H. Zhang, E. Mariani, M. D. Gao, Z. Q. Wei, X. C. Li, L. P. Han, Y. J. Shen, M. Xie, E. Cortes, D. S. Zhang, "Molecularly Engineered Co-Phthalocyanine Covalent Organic Frameworks for Efficient Nitric Oxide-to-Ammonia Electroreduction" *Journal of the American Chemical Society* **2025**, *147*, 41355-41366.

[12]	X. X. Guo, P. Wang, T. W. Wu, Z. Q. Wang, J. Li, K. Liu, J. W. Fu, M. Liu, J. Wu, Z. Lin, L. Y. Chai, Z. F. Bian, H. F. Li, M. Liu, "Aqueous Electroreduction of Nitric Oxide to Ammonia at Low Concentration via Vacancy Engineered FeOCl" *Angewandte Chemie-International Edition* **2024**, *63*.

[13]	D. D. Wang, G. L. Fan, Z. H. Pei, D. Y. Luan, X. J. Gu, X. W. D. Lou, "Efficient Cu-Co Dual-Sites in Cobalt Oxide Nanoboxes for Electrocatalytic Reduction of Low-Concentration NO to NH3" *Advanced Materials* **2025**, *37,* 2504497.

[14]	D. D. Wang, Z. W. Chen, K. Z. Gu, C. Chen, Y. Y. Liu, X. X. Wei, C. V. Singh, S. Y. Wang, "Hexagonal Cobalt Nanosheets for High-Performance Electrocatalytic NO Reduction to NH3" *Journal of the American Chemical Society* **2023**, *145*, 6899-6904.

[15]	S. Liu, L. Yan, Q. Chen, B. Wu, H. Zhu, S. Zhou, X. Wang, X. Zhao, C. Sun, Y. Li, L. Lei, Z. Li, Y. Hou, B. Yang, "Unveiling the facet-dependent activity of Cu_2_O for enhanced selective electroreduction of nitric oxide to ammonia and Zn-NO batteries" *Process Saf. Environ. Prot.* **2024**, *187*, 312-319.

[16]	B. Wu, L. Huang, L. Yan, H. Gang, Y. Cao, D. Wei, H. Wang, Z. Guo, W. Zhang, "Boron-Modulated Electronic-Configuration Tuning of Cobalt for Enhanced Nitric Oxide Fixation to Ammonia" *Nano Lett.* **2023**, *23*, 7120-7128.

[17]	Z. Wu, Y. Liu, D. Wang, Y. Zhang, K. Gu, Z. He, L. Liu, H. Liu, J. Fan, C. Chen, S. Wang, "Cu@Co with Dilatation Strain for High-Performance Electrocatalytic Reduction of Low-Concentration Nitric Oxide" *Adv. Mater.* **2024**, *36*, 2309470.

[18]	Q. Liu, Y. Lin, L. Yue, J. Liang, L. Zhang, T. Li, Y. Luo, M. Liu, J. You, A. A. Alshehri, Q. Kong, X. Sun, "Bi nanoparticles/carbon nanosheet composite: A high-efficiency electrocatalyst for NO reduction to NH_3_" *Nano Res.* **2022**, *15*, 5032-5037.

[19]	D. Wang, X. Zhu, X. Tu, X. Zhang, C. Chen, X. Wei, Y. Li, S. Wang, "Oxygen-Bridged Copper–Iron Atomic Pair as Dual-Metal Active Sites for Boosting Electrocatalytic NO Reduction" *Adv. Mater.* **2023**, *35*, 2304646.

[20]	R. Luo, B.-J. Li, Z.-P. Wang, M.-G. Chen, G.-L. Zhuang, Q. Li, J.-P. Tong, W.-T. Wang, Y.-H. Fan, F. Shao, "Two-Dimensional MOF Constructed by a Binuclear-Copper Motif for High-Performance Electrocatalytic NO Reduction to NH_3_" *JACS Au* **2024**, *4*, 3823-3832.

[21]	J. Liang, P. Y. Liu, Q. Y. Li, T. S. Li, L. C. Yue, Y. S. Luo, Q. Liu, N. Li, B. Tang, A. A. Alshehri, I. Shakir, P. O. Agboola, C. H. Sun, X. P. Sun, "Amorphous Boron Carbide on Titanium Dioxide Nanobelt Arrays for High-Efficiency Electrocatalytic NO Reduction to NH_3_" *Angewandte Chemie-International Edition* **2022**, *61,* e202202087.

[22]	J. T. Ren, L. Chen, H. Y. Wang, Z. Y. Yuan, "Aqueous Rechargeable Zn-N_2_ Battery Assembled by Bifunctional Cobalt Phosphate Nanocrystals-Loaded Carbon Nanosheets for Simultaneous NH_3_ Production and Power Generation" *Acs Applied Materials & Interfaces* **2021**, *13*, 12106-12117.

[23]	Y. F. Song, Y. H. Zhao, G. Z. Nan, W. Chen, Z. K. Guo, S. G. Li, Z. Y. Tang, W. Wei, Y. H. Sun, "Electrocatalytic oxidation of methane to ethanol via NiO/Ni interface" *Applied Catalysis B-Environmental* **2020**, *270,* 118888.
